# Supplementary material for: Hospital services utilisation and cost before and after COVID-19 hospital treatment: Evidence from Indonesia
Source: PLoS One. 2024 Jul 5;19(7):e0305835. doi: 10.1371/journal.pone.0305835 (PMC11226039; doi:10.1371/journal.pone.0305835)
Supplement: S1 Table — (PDF) [file pone.0305835.s001.pdf]

Tables

Table A1. Difference-in-Difference Estimates of Outpatient Utilization Rate of COVID-19 Group compared to Comparison Group, All Individuals

	Month in hospital with COVID-19							
	May 20		Jun. 20		Jul. 20		Aug. 20	
-9 month	0.638	(2.874)	1.198	(2.186)	0.589	(1.932)	-1.398	(1.787)
-8 month	1.506	(2.716)	2.223	(2.099)	0.889	(1.859)	0.167	(1.659)
-7 month	-0.492	(2.492)	0.557	(1.980)	0.716	(1.690)	-0.917	(1.584)
-6 month	1.966	(2.129)	0.087	(1.681)	-0.217	(1.493)	-2.041	(1.357)
-5 month	Ref.							
-4 month	1.202	(2.304)	2.696	(1.701)	2.321	(1.456)	1.421	(1.306)
-3 month	-2.883	(2.453)	4.095*	(2.010)	4.355**	(1.684)	0.197	(1.407)
-2 month	5.265	(2.801)	7.529***	(2.133)	5.778***	(1.686)	1.632	(1.554)
-1 month	7.076*	(2.787)	10.592***	(2.143)	10.097***	(1.833)	5.614**	(1.761)
**0 (COVID-19)**	20.228***	(2.921)	22.307***	(2.325)	20.380***	(2.029)	24.224***	(1.876)
+1 month	-2.729	(3.132)	1.469	(2.527)	3.252	(2.072)	2.833	(1.984)
+2 month	3.302	(3.396)	9.908***	(2.601)	5.276*	(2.140)	7.241***	(2.005)
+3 month	-1.766	(3.171)	9.615***	(2.750)	3.163	(2.148)	1.947	(2.032)
+4 month	-1.897	(3.256)	7.803**	(2.729)	1.455	(2.239)		
+5 month	-1.483	(3.328)	5.787*	(2.726)				
+6 month	-4.627	(3.370)						
Observations	188,640		221,565		255,206		251,459	
Individuals	11,790		14,771		18,229		19,343	

Note: Coefficients represented the estimated differences in number of outpatient visits per 100 individuals per month for all causes or diagnoses. Regressions control for individuals' year of birth, gender, COVID-19 severity, NHI membership segment, comorbidities prior to 2020, and month and province fixed effects. Standard errors clustered at individual level in parentheses * $p < 0.05$, ** $p < 0.01$, *** $p < 0.001$.

Table A2. Difference-in-Difference Estimates of Outpatient Utilization Rate of COVID-19 Group compared to Comparison Group, <40 years old

	Month in hospital with COVID-19							
	May 20		Jun. 20		Jul. 20		Aug. 20	
-9 month	-4.053	(2.747)	-1.243	(2.344)	3.201	(2.197)	-1.092	(2.008)
-8 month	-3.229	(2.857)	1.695	(2.271)	4.527*	(2.174)	0.226	(1.879)
-7 month	-3.070	(2.829)	0.790	(2.345)	4.985*	(1.984)	-1.657	(1.892)
-6 month	-4.409	(2.367)	3.315	(2.107)	2.168	(1.744)	-1.972	(1.590)
-5 month	Ref.							
-4 month	-4.251	(2.708)	-0.368	(1.933)	3.127	(1.622)	1.160	(1.613)
-3 month	-7.259*	(2.974)	1.783	(2.295)	2.813	(1.860)	1.476	(1.630)
-2 month	-0.470	(3.268)	1.442	(2.302)	3.764*	(1.817)	3.767*	(1.825)
-1 month	3.258	(3.230)	6.584**	(2.279)	10.960***	(2.016)	9.796***	(2.070)
**0 (COVID-19)**	21.698***	(3.253)	28.241***	(2.624)	34.734***	(2.432)	40.585***	(2.248)
+1 month	-1.547	(3.338)	3.829	(2.788)	6.641**	(2.286)	7.649***	(2.199)
+2 month	-1.955	(4.015)	2.074	(2.544)	7.176**	(2.269)	4.656*	(2.160)
+3 month	-5.569	(3.643)	3.573	(2.685)	3.768	(2.414)	1.927	(2.224)
+4 month	-5.235	(3.771)	0.427	(2.497)	1.522	(2.474)		
+5 month	-7.938*	(3.711)	-0.640	(2.755)				
+6 month	-9.651**	(3.593)						
Observations	66,736		79,710		93,338		88,582	
Individuals	4,171		5,314		6,667		6,814	

Note: Coefficients represented the estimated differences in number of outpatient visits per 100 individuals per month for all causes or diagnoses. Regressions control for individuals' year of birth, gender, COVID-19 severity, NHI membership segment, comorbidities prior to 2020, and month and province fixed effects. Standard errors clustered at individual level in parentheses * $p < 0.05$, ** $p < 0.01$, *** $p < 0.001$.

Table A3. Difference-in-Difference Estimates of Outpatient Utilization Rate of COVID-19 Group compared to Comparison Group, ≥40 years old

	Month in hospital with COVID-19							
	May 20		Jun. 20		Jul. 20		Aug. 20	
-9 month	3.962	(4.283)	2.716	(3.200)	-1.026	(2.777)	-1.335	(2.526)
-8 month	4.272	(4.006)	2.532	(3.061)	-1.193	(2.661)	0.301	(2.339)
-7 month	0.585	(3.622)	0.619	(2.819)	-1.709	(2.413)	-0.342	(2.212)
-6 month	5.784	(3.084)	-1.780	(2.367)	-1.687	(2.133)	-1.849	(1.900)
-5 month	Ref.							
-4 month	4.006	(3.308)	4.605	(2.458)	1.648	(2.102)	1.532	(1.818)
-3 month	-0.698	(3.500)	5.505	(2.884)	4.927*	(2.439)	-0.604	(1.978)
-2 month	8.414*	(4.034)	11.233***	(3.127)	6.689**	(2.455)	0.459	(2.181)
-1 month	9.184*	(4.045)	13.206***	(3.138)	9.495***	(2.661)	3.252	(2.472)
**0 (COVID-19)**	19.272***	(4.262)	19.218***	(3.354)	11.795***	(2.890)	15.806***	(2.613)
+1 month	-4.025	(4.599)	-0.218	(3.698)	1.118	(2.996)	0.452	(2.808)
+2 month	5.283	(4.873)	14.345***	(3.853)	3.987	(3.114)	8.618**	(2.861)
+3 month	0.128	(4.555)	13.437**	(4.089)	2.495	(3.099)	2.037	(2.898)
+4 month	-0.318	(4.695)	12.280**	(4.094)	1.230	(3.248)		
+5 month	1.824	(4.838)	9.671*	(4.040)				
+6 month	-1.951	(4.975)						
Observations	121,904		141,855		161,868		162,877	
Individuals	7,619		9,457		11,562		12,529	

Note: Coefficients represented the estimated differences in number of outpatient visits per 100 individuals per month for all causes or diagnoses. Regressions control for individuals' year of birth, gender, COVID-19 severity, NHI membership segment, comorbidities prior to 2020, and month and province fixed effects. Standard errors clustered at individual level in parentheses * $p < 0.05$, ** $p < 0.01$, *** $p < 0.001$.

Table A4. Difference-in-Difference Estimates of Outpatient Utilization Rate of COVID-19 Group compared to Comparison Group, Male

	Month in hospital with COVID-19							
	May 20		Jun. 20		Jul. 20		Aug. 20	
-9 month	-0.060	(4.292)	4.493	(3.110)	5.162	(2.670)	0.570	(2.513)
-8 month	3.554	(3.934)	6.136*	(2.934)	4.212	(2.543)	1.720	(2.346)
-7 month	0.405	(3.517)	4.089	(2.791)	4.232	(2.392)	0.063	(2.267)
-6 month	7.022*	(3.242)	2.585	(2.412)	0.952	(2.154)	-1.780	(1.853)
-5 month	Ref.							
-4 month	1.055	(3.357)	4.403	(2.604)	2.324	(2.075)	2.518	(1.845)
-3 month	-0.267	(3.586)	3.852	(3.037)	3.206	(2.258)	1.965	(2.041)
-2 month	6.480	(4.023)	8.465**	(3.139)	5.783*	(2.327)	2.808	(2.243)
-1 month	7.741*	(3.840)	10.396***	(3.096)	10.161***	(2.619)	8.004**	(2.641)
**0 (COVID-19)**	20.919***	(3.870)	23.928***	(3.376)	20.827***	(2.844)	25.202***	(2.730)
+1 month	-5.580	(4.340)	4.483	(3.889)	9.344***	(2.822)	6.642*	(2.906)
+2 month	9.795*	(4.761)	14.597***	(3.856)	9.219**	(3.003)	13.511***	(2.988)
+3 month	3.049	(4.385)	10.379**	(3.914)	6.529*	(2.968)	9.245**	(3.093)
+4 month	-0.279	(4.576)	9.016*	(3.833)	7.458*	(3.165)		
+5 month	2.587	(4.446)	8.485*	(3.937)				
+6 month	0.049	(4.462)						
Observations	86,832		101,985		120,064		118,534	
Individuals	5,427		6,799		8,576		9,118	

Note: Coefficients represented the estimated differences in number of outpatient visits per 100 individuals per month for all causes or diagnoses. Regressions control for individuals' year of birth, gender, COVID-19 severity, NHI membership segment, comorbidities prior to 2020, and month and province fixed effects. Standard errors clustered at individual level in parentheses * $p < 0.05$, ** $p < 0.01$, *** $p < 0.001$.

Table A5. Difference-in-Difference Estimates of Inpatient Utilization Rate of COVID-19 Group compared to Comparison Group, Female

	Month in hospital with COVID-19							
	May 20		Jun. 20		Jul. 20		Aug. 20	
-9 month	1.489	(3.866)	-1.509	(3.034)	-3.390	(2.771)	-3.153	(2.536)
-8 month	-0.509	(3.773)	-1.305	(2.961)	-1.977	(2.676)	-1.150	(2.350)
-7 month	-1.594	(3.529)	-2.628	(2.778)	-2.368	(2.381)	-1.682	(2.211)
-6 month	-2.546	(2.845)	-2.063	(2.323)	-1.261	(2.070)	-2.351	(1.964)
-5 month	Ref.							
-4 month	0.985	(3.189)	1.183	(2.224)	2.541	(2.050)	0.496	(1.844)
-3 month	-5.083	(3.388)	4.486	(2.663)	5.489*	(2.478)	-1.272	(1.948)
-2 month	4.571	(3.915)	6.773*	(2.891)	5.923*	(2.432)	0.605	(2.157)
-1 month	6.684	(4.015)	10.869***	(2.938)	10.139***	(2.569)	3.629	(2.370)
**0 (COVID-19)**	19.940***	(4.300)	20.956***	(3.177)	20.354***	(2.885)	23.627***	(2.579)
+1 month	-0.391	(4.458)	-1.194	(3.287)	-1.817	(2.995)	-0.405	(2.710)
+2 month	-2.543	(4.767)	6.094	(3.508)	1.948	(3.047)	1.635	(2.708)
+3 month	-5.914	(4.501)	8.863*	(3.821)	0.177	(3.081)	-4.468	(2.698)
+4 month	-3.612	(4.593)	6.718	(3.842)	-4.035	(3.140)		
+5 month	-5.245	(4.848)	3.555	(3.761)				
+6 month	-8.969	(4.944)						
Observations	101,808		119,580		135,142		132,925	
Individuals	6,363		7,972		9,653		10,225	

Note: Coefficients represented the estimated differences in number of Inpatient visits per 100 individuals per month for all causes or diagnoses. Regressions control for individuals' year of birth, gender, COVID-19 severity, NHI membership segment, comorbidities prior to 2020, and month and province fixed effects. Standard errors clustered at individual level in parentheses * $p < 0.05$, ** $p < 0.01$, *** $p < 0.001$.

Table A6. Difference-in-Difference Estimates of Inpatient Utilization Rate of COVID-19 Group compared to Comparison Group, All Individuals

	Month in hospital with COVID-19							
	May 20		Jun. 20		Jul. 20		Aug. 20	
-9 month	-1.060	(0.587)	-0.055	(0.477)	-0.462	(0.396)	-0.132	(0.377)
-8 month	-0.645	(0.574)	-0.011	(0.487)	-1.020*	(0.396)	-0.201	(0.373)
-7 month	-0.490	(0.578)	-0.374	(0.491)	-0.357	(0.396)	-0.421	(0.388)
-6 month	-0.410	(0.572)	0.209	(0.480)	-1.163**	(0.402)	0.224	(0.358)
-5 month	Ref.							
-4 month	-0.791	(0.633)	0.437	(0.495)	-0.428	(0.380)	-0.104	(0.330)
-3 month	0.082	(0.622)	0.646	(0.525)	0.179	(0.364)	-0.265	(0.340)
-2 month	0.611	(0.671)	0.953*	(0.459)	-0.309	(0.369)	-0.150	(0.358)
-1 month	1.385*	(0.591)	1.145*	(0.480)	0.553	(0.397)	1.213**	(0.387)
**0 (COVID-19)**	104.316***	(0.986)	103.597***	(0.778)	101.581***	(0.602)	102.746***	(0.571)
+1 month	2.556***	(0.743)	3.440***	(0.564)	2.595***	(0.430)	3.194***	(0.401)
+2 month	-0.146	(0.615)	0.588	(0.490)	0.168	(0.396)	0.462	(0.379)
+3 month	-0.437	(0.586)	0.320	(0.488)	-0.764*	(0.386)	-0.391	(0.354)
+4 month	-1.051	(0.573)	-0.312	(0.479)	-0.732*	(0.372)		
+5 month	-1.446*	(0.593)	-0.098	(0.464)				
+6 month	-0.690	(0.590)						
Observations	188,640		221,565		255,206		251,459	
Individuals	11,790		14,771		18,229		19,343	

Note: Coefficients represented the estimated differences in number of Inpatient visits per 100 individuals per month for all causes or diagnoses. Regressions control for individuals' year of birth, gender, COVID-19 severity, NHI membership segment, comorbidities prior to 2020, and month and province fixed effects. Standard errors clustered at individual level in parentheses * $p < 0.05$, ** $p < 0.01$, *** $p < 0.001$.

Table A7. Difference-in-Difference Estimates of Inpatient Utilization Rate of COVID-19 Group compared to Comparison Group, <40 years old

	Month in hospital with COVID-19							
	May 20		Jun. 20		Jul. 20		Aug. 20	
-9 month	-0.762	(0.886)	0.978	(0.726)	0.171	(0.660)	-0.259	(0.581)
-8 month	-1.134	(0.906)	1.213	(0.757)	-0.466	(0.639)	0.254	(0.558)
-7 month	-0.632	(0.960)	0.504	(0.754)	0.123	(0.630)	-0.423	(0.603)
-6 month	-0.732	(0.909)	1.149	(0.695)	-1.059	(0.640)	0.447	(0.577)
-5 month	Ref.							
-4 month	-0.915	(1.058)	0.936	(0.745)	0.041	(0.608)	-0.079	(0.497)
-3 month	-0.786	(0.944)	1.249	(0.738)	0.446	(0.590)	-0.233	(0.550)
-2 month	0.839	(1.057)	0.643	(0.653)	-0.439	(0.610)	0.019	(0.587)
-1 month	0.332	(0.909)	1.212	(0.727)	0.080	(0.612)	0.910	(0.610)
**0 (COVID-19)**	97.832^{***}	(1.557)	96.367^{***}	(1.253)	93.683^{***}	(0.979)	95.404^{***}	(0.959)
+1 month	1.801	(1.146)	3.823^{***}	(0.863)	3.253^{***}	(0.690)	4.250^{***}	(0.640)
+2 month	-0.885	(1.025)	1.253	(0.747)	0.818	(0.615)	0.345	(0.581)
+3 month	-1.179	(0.907)	1.269	(0.700)	-0.337	(0.639)	-0.023	(0.525)
+4 month	-1.066	(0.869)	0.433	(0.698)	-0.212	(0.579)		
+5 month	-2.592^{**}	(0.849)	0.774	(0.668)				
+6 month	-0.910	(0.922)						
Observations	66,736		79,710		93,338		88,582	
Individuals	4,171		5,314		6,667		6,814	

Note: Coefficients represented the estimated differences in number of Inpatient visits per 100 individuals per month for all causes or diagnoses. Regressions control for individuals' year of birth, gender, COVID-19 severity, NHI membership segment, comorbidities prior to 2020, and month and province fixed effects. Standard errors clustered at individual level in parentheses * $p < 0.05$, ** $p < 0.01$, *** $p < 0.001$.

Table A8. Difference-in-Difference Estimates of Inpatient Utilization Rate of COVID-19 Group compared to Comparison Group, ≥40 years old

	Month in hospital with COVID-19							
	May 20		Jun. 20		Jul. 20		Aug. 20	
-9 month	-1.305	(0.776)	-0.642	(0.622)	-0.789	(0.502)	0.011	(0.489)
-8 month	-0.364	(0.749)	-0.633	(0.627)	-1.264*	(0.507)	-0.440	(0.490)
-7 month	-0.320	(0.736)	-0.816	(0.637)	-0.593	(0.512)	-0.426	(0.502)
-6 month	-0.190	(0.736)	-0.284	(0.638)	-1.185*	(0.518)	0.085	(0.457)
-5 month	Ref.							
-4 month	-0.769	(0.793)	0.092	(0.649)	-0.701	(0.489)	-0.134	(0.431)
-3 month	0.531	(0.824)	0.322	(0.707)	0.069	(0.466)	-0.266	(0.432)
-2 month	0.380	(0.863)	1.159	(0.618)	-0.237	(0.467)	-0.221	(0.456)
-1 month	2.014**	(0.775)	1.188	(0.626)	0.823	(0.520)	1.371**	(0.500)
**0 (COVID-19)**	108.021***	(1.264)	107.926***	(0.991)	106.270***	(0.764)	106.825***	(0.713)
+1 month	2.925**	(0.970)	3.267***	(0.739)	2.277***	(0.556)	2.701***	(0.513)
+2 month	0.241	(0.777)	0.115	(0.634)	-0.201	(0.518)	0.544	(0.494)
+3 month	-0.055	(0.761)	-0.179	(0.651)	-1.016*	(0.489)	-0.567	(0.466)
+4 month	-0.972	(0.745)	-0.691	(0.640)	-1.030*	(0.484)		
+5 month	-0.740	(0.794)	-0.577	(0.620)				
+6 month	-0.581	(0.764)						
Observations	121,904		141,855		161,868		162,877	
Individuals	7,619		9,457		11,562		12,529	

Note: Coefficients represented the estimated differences in number of Inpatient visits per 100 individuals per month for all causes or diagnoses. Regressions control for individuals' year of birth, gender, COVID-19 severity, NHI membership segment, comorbidities prior to 2020, and month and province fixed effects. Standard errors clustered at individual level in parentheses * $p < 0.05$, ** $p < 0.01$, *** $p < 0.001$.

Table A9. Difference-in-Difference Estimates of Inpatient Utilization Rate of COVID-19 Group compared to Comparison Group, Male

	Month in hospital with COVID-19							
	May 20		Jun. 20		Jul. 20		Aug. 20	
-9 month	-1.443	(0.872)	-0.705	(0.684)	-0.228	(0.567)	-0.416	(0.533)
-8 month	-0.887	(0.855)	-0.440	(0.677)	-1.508**	(0.541)	-0.450	(0.532)
-7 month	-0.786	(0.832)	-0.382	(0.722)	-0.626	(0.554)	-0.210	(0.552)
-6 month	-0.367	(0.833)	-0.103	(0.726)	-1.001	(0.559)	0.029	(0.512)
-5 month	Ref.							
-4 month	-0.508	(0.926)	-0.171	(0.733)	-0.457	(0.528)	-0.389	(0.470)
-3 month	-0.085	(0.954)	-0.401	(0.755)	0.030	(0.517)	-0.221	(0.480)
-2 month	1.482	(1.159)	0.180	(0.670)	0.013	(0.512)	-0.465	(0.514)
-1 month	1.039	(0.903)	0.722	(0.715)	0.434	(0.553)	0.658	(0.541)
**0 (COVID-19)**	104.104***	(1.384)	104.507***	(1.155)	103.495***	(0.867)	103.700***	(0.814)
+1 month	1.477	(1.107)	2.420**	(0.829)	2.599***	(0.626)	2.724***	(0.561)
+2 month	-0.305	(0.905)	-0.209	(0.705)	0.515	(0.572)	0.105	(0.541)
+3 month	-0.662	(0.894)	-0.303	(0.688)	-1.012	(0.554)	-0.503	(0.506)
+4 month	-1.063	(0.899)	-1.323	(0.703)	-0.543	(0.525)		
+5 month	-1.514	(0.940)	-0.584	(0.678)				
+6 month	-0.391	(0.963)						
Observations	86,832		101,985		120,064		118,534	
Individuals	5,427		6,799		8,576		9,118	

Note: Coefficients represented the estimated differences in number of Inpatient visits per 100 individuals per month for all causes or diagnoses. Regressions control for individuals' year of birth, gender, COVID-19 severity, NHI membership segment, comorbidities prior to 2020, and month and province fixed effects. Standard errors clustered at individual level in parentheses * $p < 0.05$, ** $p < 0.01$, *** $p < 0.001$.

Table A10. Difference-in-Difference Estimates of Inpatient Utilization Rate of COVID-19 Group compared to Comparison Group, Female

	Month in hospital with COVID-19							
	May 20		Jun. 20		Jul. 20		Aug. 20	
-9 month	-0.797	(0.794)	0.431	(0.661)	-0.616	(0.555)	0.130	(0.528)
-8 month	-0.449	(0.771)	0.284	(0.688)	-0.560	(0.571)	0.001	(0.524)
-7 month	-0.255	(0.803)	-0.400	(0.665)	-0.143	(0.558)	-0.634	(0.545)
-6 month	-0.335	(0.791)	0.372	(0.632)	-1.312*	(0.573)	0.349	(0.501)
-5 month	Ref.							
-4 month	-1.170	(0.866)	0.926	(0.668)	-0.389	(0.540)	0.156	(0.461)
-3 month	0.163	(0.813)	1.474*	(0.723)	0.360	(0.505)	-0.293	(0.479)
-2 month	-0.242	(0.760)	1.562*	(0.619)	-0.577	(0.523)	0.111	(0.498)
-1 month	1.661*	(0.779)	1.477*	(0.644)	0.618	(0.561)	1.689**	(0.552)
**0 (COVID-19)**	104.403***	(1.394)	102.745***	(1.058)	99.927***	(0.836)	101.994***	(0.801)
+1 month	3.287***	(0.994)	4.247***	(0.773)	2.679***	(0.590)	3.626***	(0.571)
+2 month	-0.056	(0.838)	1.205	(0.676)	-0.066	(0.545)	0.757	(0.532)
+3 month	-0.356	(0.767)	0.898	(0.684)	-0.513	(0.533)	-0.297	(0.496)
+4 month	-1.121	(0.726)	0.506	(0.653)	-0.844	(0.521)		
+5 month	-1.440	(0.758)	0.284	(0.631)				
+6 month	-1.033	(0.714)						
Observations	101,808		119,580		135,142		132,925	
Individuals	6,363		7,972		9,653		10,225	

Note: Coefficients represented the estimated differences in number of outpatient visits per 100 individuals per month for all causes or diagnoses. Regressions control for individuals' year of birth, gender, COVID-19 severity, NHI membership segment, comorbidities prior to 2020, and month and province fixed effects. Standard errors clustered at individual level in parentheses * $p < 0.05$, ** $p < 0.01$, *** $p < 0.001$.
