# Supplementary material for: Hospital services utilisation and cost before and after COVID-19 hospital treatment: Evidence from Indonesia
Source: PLoS One. 2024 Jul 5;19(7):e0305835. doi: 10.1371/journal.pone.0305835 (PMC11226039; doi:10.1371/journal.pone.0305835)
Supplement: S2 Table — (PDF) [file pone.0305835.s002.pdf]

**Table B1. Outpatient Utilization Rate of COVID-19 Group and Comparison Group, All Individuals**

| Month | COVID-19 Group | | | | | | | | Comparison group | |
|---|---|---|---|---|---|---|---|---|---|---|
| | May 20 | | Jun. 20 | | Jul. 20 | | Aug. 20 | | | |
| Aug 19 | 39.91 | (2.65) | | | | | | | 43.58 | (1.48) |
| Sept 19 | 39.99 | (2.53) | 35.92 | (1.61) | | | | | 43.10 | (1.44) |
| Oct 19 | 40.45 | (2.56) | 39.92 | (1.76) | 38.13 | (1.41) | | | 45.53 | (1.50) |
| Nov 19 | 40.45 | (2.58) | 36.92 | (1.72) | 36.57 | (1.34) | 43.93 | (1.43) | 43.67 | (1.46) |
| Dec 19 | 38.13 | (2.40) | 35.80 | (1.68) | 35.99 | (1.34) | 44.80 | (1.47) | 42.92 | (1.41) |
| Jan 20 | 43.56 | (2.63) | 39.43 | (1.75) | 39.13 | (1.39) | 47.51 | (1.51) | 46.75 | (1.49) |
| Feb 20 | 38.81 | (2.43) | 40.07 | (1.75) | 37.78 | (1.32) | 45.48 | (1.44) | 45.81 | (1.49) |
| Mar 20 | 44.56 | (2.61) | 40.71 | (1.75) | 39.50 | (1.40) | 46.75 | (1.49) | 45.11 | (1.47) |
| Apr 20 | 34.94 | (2.26) | 33.25 | (1.68) | 30.25 | (1.27) | 36.19 | (1.40) | 31.27 | (1.27) |
| May 20 | **45.42** | **(1.92)** | 32.77 | (1.66) | 28.29 | (1.24) | 31.72 | (1.29) | 27.80 | (1.21) |
| June 20 | 28.86 | (1.78) | **52.57** | **(1.36)** | 39.45 | (1.39) | 40.74 | (1.45) | 36.45 | (1.39) |
| July 20 | 39.99 | (2.55) | 34.81 | (1.50) | **54.65** | **(1.13)** | 50.32 | (1.55) | 42.33 | (1.55) |
| Aug 20 | 34.66 | (2.48) | 41.35 | (1.81) | 39.07 | (1.18) | **66.91** | **(1.19)** | 40.98 | (1.48) |
| Sept 20 | 35.41 | (2.47) | 42.62 | (1.96) | 41.56 | (1.39) | 49.39 | (1.31) | 43.50 | (1.55) |
| Oct 20 | 35.12 | (2.57) | 41.39 | (1.99) | 38.40 | (1.41) | 52.18 | (1.57) | 42.41 | (1.56) |
| Nov 20 | 36.16 | (2.52) | 41.08 | (1.86) | 39.27 | (1.42) | 49.79 | (1.53) | 45.43 | (1.56) |
| Observations | 44,736 | | 92,432 | | 147,792 | | 165,584 | | 143,920 | |
| Individuals | 2,796 | | 5,777 | | 9,237 | | 10,349 | | 8,995 | |

Note: Hospital outpatient utilisation per 100 persons per month. Robust standard errors in parentheses.

**Table B2. Inpatient Utilization Rate of COVID-19 Group and Comparison Group, All Individuals**

| | COVID-19 Group | | | | | | | | Comparison group | |
|---|---|---|---|---|---|---|---|---|---|---|
| Month | May 20 | | Jun. 20 | | Jul. 20 | | Aug. 20 | | | |
| Aug 19 | 2.40 | (0.31) | | | | | | | 2.96 | (0.19) |
| Sept 19 | 2.65 | (0.34) | 2.49 | (0.22) | | | | | 2.35 | (0.17) |
| Oct 19 | 3.00 | (0.37) | 2.61 | (0.23) | 2.51 | (0.18) | | | 2.62 | (0.18) |
| Nov 19 | 3.00 | (0.36) | 2.58 | (0.23) | 2.27 | (0.17) | 3.01 | (0.19) | 2.65 | (0.18) |
| Dec 19 | 3.18 | (0.37) | 2.72 | (0.23) | 2.67 | (0.19) | 2.69 | (0.18) | 2.62 | (0.18) |
| Jan 20 | 3.61 | (0.40) | 3.36 | (0.26) | 2.73 | (0.19) | 3.20 | (0.19) | 3.22 | (0.20) |
| Feb 20 | 3.54 | (0.40) | 3.06 | (0.25) | 3.16 | (0.21) | 3.10 | (0.18) | 2.75 | (0.18) |
| Mar 20 | 4.36 | (0.44) | 3.76 | (0.29) | 3.01 | (0.19) | 3.15 | (0.19) | 2.85 | (0.19) |
| Apr 20 | 3.47 | (0.38) | 2.58 | (0.23) | 2.27 | (0.18) | 1.71 | (0.14) | 1.39 | (0.14) |
| May 20 | **107.22** | **(0.80)** | 3.08 | (0.25) | 1.86 | (0.16) | 1.77 | (0.14) | 1.72 | (0.15) |
| June 20 | 5.04 | (0.47) | **105.69** | **(0.62)** | 3.39 | (0.22) | 2.52 | (0.18) | 2.37 | (0.18) |
| July 20 | 2.75 | (0.34) | 5.11 | (0.31) | **105.58** | **(0.49)** | 3.62 | (0.22) | 2.31 | (0.18) |
| Aug 20 | 2.43 | (0.33) | 2.42 | (0.23) | 5.00 | (0.25) | **105.14** | **(0.50)** | 2.00 | (0.16) |
| Sept 20 | 1.97 | (0.30) | 2.30 | (0.22) | 2.49 | (0.19) | 5.59 | (0.24) | 1.99 | (0.17) |
| Oct 20 | 1.65 | (0.28) | 1.97 | (0.20) | 2.00 | (0.17) | 2.97 | (0.20) | 2.20 | (0.18) |
| Nov 20 | 2.00 | (0.30) | 1.92 | (0.20) | 1.93 | (0.16) | 2.09 | (0.16) | 2.13 | (0.16) |
| Observations | 44,736 | | 92,432 | | 147,792 | | 165,584 | | 143,920 | |
| Individuals | 2,796 | | 5,777 | | 9,237 | | 10,349 | | 8,995 | |

Note: Hospital inpatient utilisation per 100 persons per month. Robust standard errors in parentheses.

**Table B3. Average Hospital Cost of COVID-19 Group and Comparison Group, All Individuals**

| Month | COVID-19 Group | | | | | | | | Comparison group | |
|---|---|---|---|---|---|---|---|---|---|---|
| | May 20 | | Jun. 20 | | Jul. 20 | | Aug. 20 | | | |
| Aug 19 | 288 | (35) | 281 | (20) | 260 | (16) | 338 | (17) | 342 | (23) |
| Sept 19 | 283 | (29) | 262 | (20) | 249 | (17) | 318 | (20) | 298 | (21) |
| Oct 19 | 290 | (28) | 291 | (23) | 310 | (24) | 343 | (18) | 309 | (19) |
| Nov 19 | 320 | (41) | 283 | (21) | 264 | (16) | 359 | (22) | 302 | (17) |
| Dec 19 | 340 | (41) | 284 | (21) | 278 | (16) | 346 | (25) | 297 | (16) |
| Jan 20 | 347 | (34) | 354 | (27) | 293 | (16) | 373 | (19) | 364 | (22) |
| Feb 20 | 340 | (34) | 296 | (19) | 327 | (19) | 361 | (22) | 303 | (15) |
| Mar 20 | 364 | (34) | 362 | (26) | 330 | (20) | 374 | (19) | 323 | (18) |
| Apr 20 | 338 | (36) | 288 | (22) | 260 | (16) | 273 | (17) | 210 | (16) |
| May 20 | **169,912** | **(2,163)** | 311 | (24) | 227 | (14) | 266 | (19) | 203 | (14) |
| June 20 | 2,393 | (352) | **138,787** | **(1,212)** | 377 | (27) | 330 | (17) | 272 | (16) |
| July 20 | 890 | (162) | 2,200 | (223) | **108,349** | **(771)** | 417 | (19) | 312 | (21) |
| Aug 20 | 529 | (101) | 551 | (67) | 1,641 | (143) | **98,703** | **(718)** | 295 | (20) |
| Sept 20 | 713 | (169) | 576 | (79) | 520 | (47) | 1,443 | (110) | 306 | (18) |
| Oct 20 | 426 | (78) | 388 | (40) | 410 | (35) | 671 | (56) | 312 | (18) |
| Nov 20 | 393 | (72) | 472 | (55) | 475 | (48) | 465 | (34) | 313 | (17) |
| Observations | 44,736 | | 92,432 | | 147,792 | | 165,584 | | 143,920 | |
| Individuals | 2,796 | | 5,777 | | 9,237 | | 10,349 | | 8,995 | |

Note: Average monthly hospital cost in thousand rupiah. Robust standard errors in parentheses.
