# Supplementary material for: Hospital services utilisation and cost before and after COVID-19 hospital treatment: Evidence from Indonesia
Source: PLoS One. 2024 Jul 5;19(7):e0305835. doi: 10.1371/journal.pone.0305835 (PMC11226039; doi:10.1371/journal.pone.0305835)

PLOS ONE

Hospital services utilisation and cost before and after COVID-19 hospital treatment: evidence from Indonesia

--Manuscript Draft--

Manuscript Number:	PONE-D-22-31470
Article Type:	Research Article
Full Title:	Hospital services utilisation and cost before and after COVID-19 hospital treatment: evidence from Indonesia
Short Title:	Hospital services utilisation and cost before and after COVID-19 hospital treatment
Corresponding Author:	Firdaus Hafidz, M.D., MPH. PhD<br>Gadjah Mada University Faculty of Medicine, Public Health, and Nursing: Universitas Gadjah Mada Fakultas Kedokteran Kesehatan Masyarakat dan Keperawatan<br>Sleman, Yogyakarta INDONESIA
Keywords:	COVID-19; hospital treatment; cost; difference-in-difference; Indonesia
Abstract:	Objective: To estimate hospital services utilisation and cost among the Indonesian population enrolled in the National Health Insurance (NHI) program before and after COVID-19 hospital treatment.<br>Methods: 28,159 Indonesian NHI enrollees treated with laboratory-confirmed COVID-19 in hospitals between May and August 2020 were compared to 8,995 individuals never diagnosed with COVID-19 in 2020. A difference-in-difference approach is used to contrast the monthly all-cause utilisation rate and total claims of hospital services between these two groups. A period of nine months before and three to six months after hospital treatment were included in the analysis.<br>Results: A substantial short-term increase in hospital services utilisation and cost before and after COVID-19 treatment was observed. Using the fifth month before treatment as the reference period, we observed an increased outpatient visits rate in 1-3 calendar months before and up to 2-4 months after treatment (p<0.001) among the COVID-19 group compared to the control. We also found a higher admissions rate in 1-2 months before and one month after treatment (p<0.001). Consequently, increased hospital costs were observed in 1-3 calendar months and 1-4 calendar months after the treatment (p<0.001). The elevated hospital resource utilisation was more prominent among individuals older than 40. Overall, no substantial increase in hospital outpatient visits, admissions, and costs beyond four months after and five months before COVID-19 treatment.<br>Conclusion: Individuals with COVID-19 who required hospital treatment had considerably higher healthcare resource utilisation in the short-term, before and after the treatment. These findings indicated that the total cost of treating COVID-19 patients might include the pre and post-acute period.
Order of Authors:	Muhammad Fikru Rizal<br>Firdaus Hafidz, M.D., MPH. PhD<br>Gilbert Renardi Kusila<br>Wan Aisyiah<br>Dedy Revelino<br>Erzan Dhanalvin<br>Ayunda Oktavia<br>Ilyasa Ilyasa<br>Mahlil Ruby<br>Benyamin Saut<br>Citra Jaya
Additional Information:	

Powered by Editorial Manager® and ProduXion Manager® from Aries Systems Corporation

Question	Response
**Financial Disclosure**<br><br>Enter a financial disclosure statement that describes the sources of funding for the work included in this submission. Review the submission guidelines for detailed requirements. View published research articles from *PLOS ONE* for specific examples.<br><br>This statement is required for submission and **will appear in the published article** if the submission is accepted. Please make sure it is accurate.<br><br>**Unfunded studies**<br>Enter: *The author(s) received no specific funding for this work.*<br><br>**Funded studies**<br>Enter a statement with the following details:<br>• Initials of the authors who received each award<br>• Grant numbers awarded to each author<br>• The full name of each funder<br>• URL of each funder website<br>• Did the sponsors or funders play any role in the study design, data collection and analysis, decision to publish, or preparation of the manuscript?<br>• **NO** - Include this sentence at the end of your statement: *The funders had no role in study design, data collection and analysis, decision to publish, or preparation of the manuscript.*<br>• **YES** - Specify the role(s) played.<br><br>* typeset	This study is fully funded by Indonesia's Social Security Administrative Body of Health (BPJS-Kesehatan). Award/grant number 456/BA/0621
**Competing Interests**<br><br>Use the instructions below to enter a competing interest statement for this submission. On behalf of all authors, disclose any competing interests that could be perceived to bias this work—acknowledging all financial support and any other relevant financial or non-financial competing interests.	The authors have declared that no competing interests exist

This statement is required for submission and **will appear in the published article** if the submission is accepted. Please make sure it is accurate and that any funding sources listed in your Funding Information later in the submission form are also declared in your Financial Disclosure statement.<br><br>View published research articles from *PLOS ONE* for specific examples.<br><br>**NO authors have competing interests**<br><br>Enter: *The authors have declared that no competing interests exist.*<br><br>**Authors with competing interests**<br><br>Enter competing interest details beginning with this statement:<br><br>*I have read the journal's policy and the authors of this manuscript have the following competing interests: [insert competing interests here]*<br><br>* typeset	
**Ethics Statement**<br><br>Enter an ethics statement for this submission. This statement is required if the study involved:<br><br>• Human participants<br>• Human specimens or tissue<br>• Vertebrate animals or cephalopods<br>• Vertebrate embryos or tissues<br>• Field research<br><br>Write "N/A" if the submission does not require an ethics statement.<br><br>General guidance is provided below. Consult the submission guidelines for detailed instructions. **Make sure that all information entered here is included in the Methods section of the manuscript.**	This study obtained ethical clearance from the Ethical Committee of the Faculty of Medicine, Public Health, and Nursing, Universitas Gadjah Mada No KE/FK/0945/EC/2021.

Powered by Editorial Manager® and ProduXion Manager® from Aries Systems Corporation

**Format for specific study types**<br><br>**Human Subject Research (involving human participants and/or tissue)**<br>• Give the name of the institutional review board or ethics committee that approved the study<br>• Include the approval number and/or a statement indicating approval of this research<br>• Indicate the form of consent obtained (written/oral) or the reason that consent was not obtained (e.g. the data were analyzed anonymously)<br><br>**Animal Research (involving vertebrate animals, embryos or tissues)**<br>• Provide the name of the Institutional Animal Care and Use Committee (IACUC) or other relevant ethics board that reviewed the study protocol, and indicate whether they approved this research or granted a formal waiver of ethical approval<br>• Include an approval number if one was obtained<br>• If the study involved *non-human primates*, add *additional details* about animal welfare and steps taken to ameliorate suffering<br>• If anesthesia, euthanasia, or any kind of animal sacrifice is part of the study, include briefly which substances and/or methods were applied<br><br>**Field Research**<br><br>Include the following details if this study involves the collection of plant, animal, or other materials from a natural setting:<br>• Field permit number<br>• Name of the institution or relevant body that granted permission	
**Data Availability**<br><br>Authors are required to make all data underlying the findings described fully available, without restriction, and from the time of publication. PLOS allows rare exceptions to address legal and ethical concerns. See the PLOS Data Policy and FAQ for detailed information.	No - some restrictions will apply

A Data Availability Statement describing where the data can be found is required at submission. Your answers to this question constitute the Data Availability Statement and **will be published in the article**, if accepted.<br><br>**Important:** Stating 'data available on request from the author' is not sufficient. If your data are only available upon request, select 'No' for the first question and explain your exceptional situation in the text box.<br><br>Do the authors confirm that all data underlying the findings described in their manuscript are fully available without restriction?	
**Describe where the data may be found in full sentences. If you are copying our sample text, replace any instances of XXX with the appropriate details.**<br><br>• If the data are **held or will be held in a public repository**, include URLs, accession numbers or DOIs. If this information will only be available after acceptance, indicate this by ticking the box below. For example: *All XXX files are available from the XXX database (accession number(s) XXX, XXX.*).<br>• If the data are all contained **within the manuscript and/or Supporting Information files**, enter the following: *All relevant data are within the manuscript and its Supporting Information files.*<br>• If neither of these applies but you are able to provide **details of access elsewhere**, with or without limitations, please do so. For example:<br><br>*Data cannot be shared publicly because of [XXX]. Data are available from the XXX Institutional Data Access / Ethics Committee (contact via XXX) for researchers who meet the criteria for access to confidential data.*<br><br>*The data underlying the results presented in the study are available*	Data cannot be shared publicly because of managed by the Social Security Administrative Body of Health (BPJS-Kesehatan) and only available on a reasonable request. Data are available from the Social Security Administrative Body of Health (BPJS-Kesehatan) (contact via https://e-ppid.bpjs-kesehatan.go.id/eppid/#/home/beranda) for researchers who meet the criteria for access to confidential data.

Powered by Editorial Manager® and ProduXion Manager® from Aries Systems Corporation

from (include the name of the third party and contact information or URL).<br>• This text is appropriate if the data are owned by a third party and authors do not have permission to share the data.<br><br>* typeset	
Additional data availability information:	

Manuscript

Click here to access/download;Manuscript;Main Text
Manuscript_fh_mfr_wopic.docx

Hospital services utilisation and cost before and after COVID-19 hospital treatment: evidence from Indonesia

Muhammad Fikru Rizal[1,3], Firdaus Hafidz[1*], Gilbert Renardi Kusila[1], Wan Aisyiah[2], Dedy Revelino[2], Erzan Dhanalvin[2], Ayunda Oktavia[2], Ilyasa[2], Mahlil Ruby[2], Benyamin Saut[2], Citra Jaya[2]

[1] Faculty of Medicine, Public Health, and Nursing, Universitas Gadjah Mada, Indonesia

[2] Centre for Health Economics, Monash Business School, Monash University, Australia

[3] Badan Penyelenggara Jaminan Sosial Kesehatan (Social Security Administrative Body of Health), Indonesia

* Corresponding author

E-mail: hafidz.firdaus@ugm.ac.id

Contributorship statement

MFR, FH, GRK: Conceptualisation, Methodology, Data Management, Data Analysis, Writing – Original Draft Preparation, Writing - Review & Editing

WA, DR, ED, BS: Conceptualisation, Data Management, Reviewing

AO, I, MR, CJ: Data Collection, Writing – Review & Editing,

Abstract

Objective: To estimate hospital services utilisation and cost among the Indonesian population enrolled in the National Health Insurance (NHI) program before and after COVID-19 hospital treatment.

Methods: 28,159 Indonesian NHI enrollees treated with laboratory-confirmed COVID-19 in hospitals between May and August 2020 were compared to 8,995 individuals never diagnosed with COVID-19 in 2020. A difference-in-difference approach is used to contrast the monthly all-cause utilisation rate and total claims of hospital services between these two groups. A period of nine months before and three to six months after hospital treatment were included in the analysis.

Results: A substantial short-term increase in hospital services utilisation and cost before and after COVID-19 treatment was observed. Using the fifth month before treatment as the reference period, we observed an increased outpatient visits rate in 1-3 calendar months before and up to 2-4 months after treatment ($p<0.001$) among the COVID-19 group compared to the control. We also found a higher admissions rate in 1-2 months before and one month after treatment ($p<0.001$). Consequently, increased hospital costs were observed in 1-3 calendar months and 1-4 calendar months after the treatment ($p<0.001$). The elevated hospital resource utilisation was more prominent among individuals older than 40. Overall, no substantial increase in hospital outpatient visits, admissions, and costs beyond four months after and five months before COVID-19 treatment.

Conclusion: Individuals with COVID-19 who required hospital treatment had considerably higher healthcare resource utilisation in the short-term, before and after the treatment. These findings indicated that the total cost of treating COVID-19 patients might include the pre and post-acute period.

Introduction

As the COVID-19 pandemic continues to become a global health issue, the understanding of its consequences beyond the respiratory system and the acute treatment for the disease itself has become a major concern. Several studies found that COVID-19 infection does not only affect the respiratory system but also hematological, cardiovascular, neurological, and gastrointestinal organs [1]. It has also been found that older people and those with chronic illnesses are more vulnerable to developing severe and critical COVID-19 infections. Moreover, these groups also have a higher mortality rate [2]. A study in the US also mentioned that COVID-19 hospitalisation among patients with preexisting comorbidities was six times higher compared to those without ones [3]. The same pattern also emerged from multiple settings, including in low-and-middle-income countries (LMICs) [4].

In addition, our understanding of the clinical sequelae of COVID-19 infections has also grown over time. Due to its multi-organ involvement, some COVID-19 symptoms can persist up to six months after infection remission or more, defined as long covid or post-covid syndrome [5]. A meta-analysis conducted by Chen et al. found that fatigue, memory problem, and respiratory issue are the three most prevalent symptoms [6]. Current evidence indicates that older patients, those with more than five symptoms and hospitalised, and the Asian population are also identified to be more likely to experience long covid [5-7].

The daily activities and quality of life deteriorate among the people who suffer from long covid, which physical limitations and emotional stress included [8, 9]. The post-covid examination and therapies are then needed in a large proportion of recovered patients. Furthermore, routine pulmonary rehabilitation and some medication consumptions were marked effective in decreasing dyspnea among long covid patients [10]. From a policymaker's perspective, the importance of long-covid cannot be understated. In the short run, the health system in each country needs to anticipate the potential change in healthcare needs through better allocation of resources and investment [11]. However, evidence on the potential impact of COVID-19 infections on subsequent healthcare use and costs is scarce, especially in low-resource settings.

Findings from National Health Services (NHS) in England indicate that COVID-19 hospitalisation increases the readmission rate and subsequent diagnosis of respiratory diseases, diabetes, and cardiovascular diseases [12]. For mild infections, a study in Norway found a short-term elevation of primary care use, mostly for a respiratory and general or unspecified condition, but no effect on specialist care. This change, however, mostly reverted after three

months [13, 14]. While these studies used a large administrative database, hence minimising reporting bias, none of them reported the costs associated with pre- or post-COVID changes in healthcare use.

This paper aimed to fill the literature gap by estimating the change in utilisation and cost of hospital services before and after individuals were treated in hospitals with laboratory-confirmed COVID-19. We leveraged administrative National Health Insurance (NHI) data from Indonesia, a lower-middle-income country (LMIC) with about 260 million population. The Indonesian NHI is the biggest single-payer social health insurance program in the world, covering more than 90% of the Indonesian population [15]. We explored the short to medium-term change in the pre- and post-acute period of COVID-19 treatment to fully understand the extent to which COVID-19 might associated with the use of hospital services, especially from a resource-constrained setting.

Methods

Data Source

We utilised all COVID-19-related hospital claims which were recorded in the deidentified National Health Insurance (NHI) database from January 2020 to February 2021. Patients who were in this COVID-19 claims data were linked with their regular hospital claims records dated back to December 2016. The merged dataset contains information related to patients' basic demographic characteristics and healthcare utilisation records. The basic demographic characteristics include the year of birth, sex, district and province where individuals were treated with COVID-19 and type of NHI membership (subsidised or non-subsidised scheme)

The utilisation records contain the month of encounter, type of services (inpatient or outpatient), primary and secondary diagnoses, diagnoses-related group codes (used as the basis for hospital payment), levels of hospitals (a basic type D hospital to an advanced type A hospital), amount of claims, and the discharge status (in-hospital death, routine, or against medical advice), and COVID-19 diagnosis status (suspect, probable, or laboratory-confirmed).

Study Population

Individuals who were diagnosed based on RT-PCR results and treated, either in outpatient care or admitted to inpatient care from May to August 2020 were included in the *COVID-19 group*. Individuals who were under monitoring and recorded as suspect or probable cases of COVID-19 in the previous months before laboratory confirmation were excluded. Similarly, those who

died during COVID-19 hospitalisation or in subsequent months were also excluded from the analysis. Those who treated in hospitals with COVID-19 in February 2021 were classified as the *control group*, assuming they never contracted COVID-19 in 2020.

Study period

We considered a period of August 2019 to November 2020 in our analysis. December 2020 and January 2021 were excluded due to the possibility of elevated healthcare utilisation two months before individuals in the control group were hospitalised with COVID-19 in February 2021. Hence, we had a maximum of six months (June 2020 to November 2020) and a minimum of three months (September to November 2020) post-COVID-19 follow-up. For pre-COVID period, we included nine months before COVID-19 hospital treatment (see Fig 1).

Fig 1. Observation period for COVID-19 and control group. Note: This figure illustrates the grouping strategy and the period of observation used in our analyses

Outcome Variables

We measured the total number of hospital service utilisation, both inpatient and outpatient, and the total claims (in Indonesian Rupiahs) separately for COVID-19 diagnosis and other diagnoses. All these measures were recorded for each individual on a monthly basis. During the month of COVID-19 diagnosis and treatment, all the patients would have at least one hospital encounter.

Statistical Analysis

Baseline characteristics of the COVID-19 group (overall and separately by the month of treatment) and the control group were described as percentages (for categorical variables) or mean and standard deviation (for continuous variable).

Next, we employed a difference-in-difference (DiD) event-study approach as implemented by Skyrud et al. and Magnusson et al. to estimate the association between COVID-19 hospital treatment and the utilisation and costs of hospital services, before and after the treatment [13, 14]. This technique estimated the association between an exposure and an outcome by contrasting the change in outcome among the exposed group from a given time before exposure (the reference period) to another time point with the change in outcomes among the control group within the same time frame. To identify causal effect of an exposure, it is assumed that

in the absence of exposure, there should be no difference in outcomes trends over time between the exposed (COVID-19) group and the unexposed (control) group [16, 17].

Empirically, we estimated the following regression model.

$$Y_{it} = \alpha_i + \delta_t + \Sigma_{t=Min}^{t=Max}\,\beta_t\,Month_t \times Covid_i + Covid_i + Month_t + X_i + \varepsilon_i \qquad (1)$$

The Y_{it} variable represents the outcomes of individuals (i) in the month (t). The variable X_i is a control variable that includes gender, year of birth, history of comorbidities, NHI membership scheme, and province fixed effects. *Month* is a categorical variable indicating the month or period of observation, with month *zero* indicating the month of laboratory-confirmed COVID-19 treatment. Min and Max indicate how many months (or groups of months) before and after COVID-19 hospitalisation, respectively. $Covid\_i$ is a binary variable indicating whether individuals are in the COVID-19 or in the control group. As previously discussed, nine months period before exposure and three to six months period after exposure were used in the main analyses. Coefficient β_t capture the month specific DiD estimates measuring the association between COVID-19 hospital treatment and changes in each of the outcomes (Equation 1).

Results

Study Participants

Of 247,650 NHI members with COVID-19-related hospital encounters between January 2020 and February 2021, 146,240 were laboratory-confirmed, while the others were categorised as suspect or probable cases only. We excluded those who died during or after COVID-19 treatment and those who were hospitalised as suspect or probable COVID-19 cases in the previous month before laboratory confirmation (see Fig 2). Our final sample consisted of 28,159 individuals in the COVID-19 group (those in hospitals with COVID-19 between May and August 2020) and 8,995 individuals in the control group (those in hospitals with COVID-19 in February 2021).

Fig 2. Study participants flow. Note: This figure depicts the flow of study participants and their classification into COVID-19 and control group.

The average age of our sample (per January 2020) ranged from 44.6 to 46.5 years old, with some variations between groups. There were slightly more females (51.7% to 54.2%) and substantially higher individuals who were non-subsidised members of the NHI (81% to 85.9%). The subsidised scheme of the NHI, either provided by the central or regional government, was intended to target the poorer population. Most of the study population were from Java and Bali

region (58.2% for May 2020 group to 80.7% in the Control group). Lastly, hypertension (11.1% to 14.6%) and diabetes (10.9% to 13%) were the two most common pre-COVID-19 comorbid – defined as diagnoses received by individuals before January 2020 (see Table 1).

Table 1. Baseline characteristics of COVID-19 cases treated in hospitals in Indonesia from May to August 2020 compared with the control group

	COVID-19 group – Month in hospital with COVID-19					Control group
	May 20	Jun. 20	Jul. 20	Aug. 20	All	
Age, mean (SD)	44.6 (15.8)	44.5 (15.8)	44.2 (15.6)	45.4 (15.6)	44.7 (15.6)	46.5 (16.2)
Age group, %						
<20	4.3	4.1	4.2	3.8	4.0	4.3
20 – 39	34.8	34.6	34.7	32.4	33.9	29.9
40 – 59	42.3	42.4	44.5	45.1	44.1	42.9
≥40	18.6	18.8	16.6	18.8	18.1	22.9
Sex, %						
Male	46.9	46.4	48.3	48.3	47.8	45.8
Female	53.1	53.6	51.7	51.7	52.2	54.2
Membership scheme, %						
Subsidised	19.0	18.3	16.2	17.0	17.2	14.1
Non-subsidized	81.0	81.7	83.8	83.0	82.8	85.9
Comorbidity, %						
Hypertension	11.1	12.7	11.6	12.7	12.2	14.6
Diabetes	10.9	11.8	11.2	12.3	11.7	13.0
Heart disease	6.2	7.2	6.4	7.4	6.9	8.2
Tuberculosis	2.3	2.1	1.8	1.8	1.9	2.3
Asthma	2.4	2.0	2.4	2.3	2.3	2.4
COPD	2.0	1.6	1.3	1.5	1.5	1.8
Cancer	4.1	4.4	3.9	4.4	4.2	4.6
Liver disease	1.8	1.4	1.3	1.3	1.4	1.6
Kidney failure	2.7	2.7	2.6	3.2	2.8	2.9
Region, %						
Sumatera	13.8	9.2	9.6	12.6	11.0	8.7
Java-Bali	58.3	63.4	68.9	73.0	68.2	80.7
Kalimantan	7.3	7.7	7.5	7.4	7.5	4.6
Sulawesi	13.5	12.7	7.1	3.2	7.5	1.0
Nusa Tenggara, Maluku, and Papua	6.4	6.4	5.4	2.7	4.7	4.7
Observations	2,796	5,777	9,237	10,349	28,159	8,995

Note: COVID-19 group were divided into month based on their initial laboratory-confirmed diagnoses that were recorded in the COVID-19 hospital claims dataset. Individuals without any hospital encounter before January 2020 or who died of any causes were excluded. Control group consisted of individuals who were diagnosed and treated in hospital with COVID-19 in February 202. Individuals' age was in January 2020.

Changes in Utilisation of Hospital Services

Fig 3 presented the difference-in-difference event-study estimates in outpatient visits rate of the COVID-19 group compared with the control group separately by treatment month. We presented the results for all ages, younger (<40 years old) and older (≥40 years old) individuals. The fifth month leading to the COVID-19 event was used as the reference period. We assumed the difference in outcome between the case and control in other periods to be constant. This assumption was confirmed by the fact that there was no statistically significant difference in the trend of outpatient or inpatient utilisation rates between the two groups. This pattern was also true for both younger and older individuals.

Fig 3. Difference-in-Difference Estimates of Hospital Outpatient Visit Rate of COVID-19 Group compared to Control Group. Note: The outcome is the number of inpatient stays per 100 individuals per month. The horizontal axis represented months relative to COVID-19 hospital treatment. Lines represented the estimated difference between the COVID-19 and control groups, controlling for demographic characteristics. Shaded areas represented the 95% Confidence Intervals.

Fig 4. Difference-in-Difference Estimates of Hospital Inpatient Rate of COVID-19 Group compared to Control Group. Note: The outcome variable is the number of inpatient stays per 100 individuals per month. The horizontal axis represented months relative to COVID-19 hospital treatment. Lines represented the estimated difference between the COVID-19 and control groups, controlling for demographic characteristics. Shaded areas represented the 95% Confidence Intervals. Estimates for the month during laboratory-confirmed COVID-19 in hospital treatment were omitted because almost all individuals in the COVID-19 group had at least one hospital encounter.

Starting from three months before treatment month, we observed some statistically significant increases in outpatient utilisation rate that peaked at the month of treatment with about 20 more visits per 100 individuals per month (p-value <0.001). In the subsequent months, those in hospitals with COVID-19 from June to August were still using more outpatient visits in about two to three months. A similar pattern was not observed for those who got COVID-19 in May. Further analysis indicated that this post-COVID trend was driven mainly by the older population. As for inpatient utilisation, overall, we only observed a significant increase of about 21 to 33 additional admissions per 1,000 individuals ($p < 0.001$) at one month before and after COVID-19 (Fig 4).

Changes in Total Costs of Hospital Services

Tables 2 to 4 presented the estimated difference-in-difference event-study in total costs of hospital services of the COVID-19 group compared with the control group, separately for each month when individuals were in hospitals with COVID-19. Results for all ages were shown in Table 2, while results for the younger and older group were provided in Table 3 and 4, respectively. In our regression, we used the period of 6 to 4 months before COVID-19 hospitalisation as the reference time where we assume no difference in admissions rate between the COVID-19 and the control group. We also lumped together 9 to 7 months before COVID-19 to see whether there was any gap in outcomes between the two groups.

We found a statistically significant increase of about 113 to 118 thousand Rupiahs in monthly hospital costs one month before the month of COVID-19 treatment (p<0.01), except for those who were treated in August 2020. In addition, those who were treated in June and July seemed to have higher monthly hospital costs starting from three months (for those treated in July) or two months (for those treated in June). Compared to the difference at the reference time (month -4 to -6), we found no statistically significant gap in inpatient use in the previous months

(month -7 to -9) between COVID-19 and the control group. This finding indicated that the two groups were relatively similar beyond six months before COVID-19 treatment.

During the month of treatment, the NHI expensed about 98 to 169 million Rupiahs more to those with COVID-19 than the control group ($p<0.001$). The range for individuals aged 40 years or older was higher, with about 111 to 191 million Rupiahs ($p<0.001$). Afterwards, we still observed greater monthly hospital claims up to five months, with a decreased amount over time. The differences ranged between 1 to 2 million rupiahs in the first month ($p<0.001$) to about 100 to 170 thousand rupiahs in the fifth month after COVID-19 treatment (although it is only statistically significant with $p<0.01$ for those who were treated in June 2020). Our analysis by age group revealed that the higher post-Covid hospital claims were concentrated in the older group (see Tables 3 and 4).

Table 2. Difference-in-Difference Estimates of Total Costs of Hospital Services of COVID-19 Group compared to Control Group, All Ages

	Month in hospital with COVID-19							
	May 20		Jun. 20		Jul. 20		Aug. 20	
-7 to -9 month	-43.4	(28.5)	-14.1	(20.1)	-5.65	(18.1)	-19.5	(19)
-4 to -6 month	Reference							
-3 month	22.8	(34.6)	49.5	(27.4)	62.8**	(21.9)	5.16	(19.2)
-2 month	26.7	(36.4)	87.9***	(24.9)	36.2	(19.4)	.597	(20.8)
-1 month	113**	(38.9)	118***	(26.9)	118***	(30.1)	47.7	(24.9)
**0 (COVID-19)**	**169,694*****	**(2,161)**	**138,526*****	**(1,211)**	**108,050*****	**(770)**	**98,351*****	**(717)**
+1 month	2,107***	(353)	1,899***	(223)	1,360***	(144)	1,080***	(111)
+2 month	563***	(160)	266***	(69.5)	228***	(49.3)	302***	(57.6)
+3 month	219*	(103)	280***	(80.4)	112**	(39.3)	94.3*	(37.6)
+4 month	393*	(169)	86.1*	(43.7)	175***	(50.6)		
+5 month	100	(81)	169**	(58)				
+6 month	66.4	(73.4)						
Observations	251,598		293,517		336,152		341,399	
Individuals	11,790		14,771		18,229		19,343	

Note: Coefficients represented the estimated differences in total costs of hospital services (in thousand Rupiahs) for both outpatient and inpatient and for all causes or diagnoses. Regressions control for individuals' year of birth, gender, COVID-19 severity, NHI membership segment, comorbidities prior to 2020, and month and province fixed effects. Standard errors clustered at individual level in parentheses * $p < 0.05$, ** $p < 0.01$, *** $p < 0.001$.

Table 3. Difference-in-Difference Estimates of Total Costs of Hospital Services of COVID-19 Group compared to Control Group, Age <40

	Month in hospital with COVID-19							
	May 20		Jun. 20		Jul. 20		Aug. 20	
-7 to -9 month	-19.7	(28.1)	35.4	(21.5)	23.4	(19.8)	-6.24	(21.4)
-4 to -6 month	Reference							
-3 month	-45.8	(38.3)	71.8*	(30.1)	18	(38.2)	-1.22	(31.6)
-2 month	28.4	(41.5)	4.03	(38.6)	-22.3	(30.1)	11.9	(26.4)
-1 month	47.6	(60.6)	27	(32.6)	83.6	(47.6)	43.8	(29.9)
**0 (COVID-19)**	**139,075*****	**(3,038)**	**111,053*****	**(1,653)**	**87,496*****	**(1,036)**	**76,383*****	**(960)**
+1 month	1,217**	(378)	1,322***	(249)	947***	(155)	786***	(126)
+2 month	244	(175)	222*	(101)	215**	(71.3)	261**	(82.7)
+3 month	149	(106)	237*	(105)	105	(58.2)	84.8	(53.2)
+4 month	258	(200)	32.2	(42.6)	150*	(76.3)		
+5 month	96.7	(130)	201	(103)				
+6 month	-76.7	(57.1)						
Observations	91,965		108,660		125,943		124,380	
Individuals	4,350		5,540		6,942		7,110	

Note: Coefficients represented the estimated differences in total costs of hospital services (in thousand Rupiahs) for both outpatient and inpatient and for all causes or diagnoses. Regressions control for individuals' year of birth, gender, COVID-19 severity, NHI membership segment, comorbidities prior to 2020, and month and province fixed effects. Standard errors clustered at individual level in parentheses * $p < 0.05$, ** $p < 0.01$, *** $p < 0.001$.

Table 4. Difference-in-Difference Estimates of Total Costs of Hospital Services of COVID-19 Group compared to Control Group, Age ≥ 40

	Month in hospital with COVID-19							
	May 20		Jun. 20		Jul. 20		Aug. 20	
-7 to -9 month	-56.7	(43.6)	-44.4	(29.6)	-24.1	(26.7)	-25.6	(27.5)
-4 to -6 month	Reference							
-3 month	66.5	(52.2)	36.4	(40.5)	83.8**	(27.2)	5.35	(24.2)
-2 month	26.4	(54.1)	136***	(33.7)	63.7*	(25.8)	-6.59	(29.1)
-1 month	148**	(51.5)	168***	(39.9)	138***	(38.3)	50.8	(35.4)
**0 (COVID-19)**	**191,230*****	**(2,883)**	**157,293*****	**(1,620)**	**122,082*****	**(1,045)**	**111,717*****	**(956)**
+1 month	2,726***	(538)	2,291***	(335)	1,638***	(218)	1,258***	(161)
+2 month	785**	(242)	292**	(94.2)	232***	(67.2)	327***	(77.7)
+3 month	263	(158)	306**	(115)	113*	(52.6)	99.9	(51.2)
+4 month	483	(251)	120	(67.2)	188**	(67.1)		
+5 month	98.1	(103)	143*	(67.3)				
+6 month	162	(118)						
Observations	159,633		184,857		210,209		217,019	
Individuals	7,440		9,231		11,287		12,233	

Note: Coefficients represented the estimated differences in total costs of hospital services (in thousand Rupiahs) for outpatient and inpatient services (all-cause). Regressions control for individuals' birth year, gender, COVID-19 severity, NHI membership segment, comorbidities before 2020, and month and province fixed effects. Standard errors clustered at individual level in parentheses * $p < 0.05$, ** $p < 0.01$, *** $p < 0.001$.

Discussion

Main findings

Among 28,155 Indonesian National Health Insurance (NHI) enrollees who were in hospitals with laboratory-confirmed COVID-19 between May and August 2020, our results indicated some elevated uses and costs of hospital services months before and after the treatment month. Specifically, we found an increased outpatient utilisation that happened three months before and up to three months after the treatment and an uptick in admissions rate one month before and after the treatment. These observed post-COVID trends were consistent with a previous study in England that found a higher readmission rate among discharged COVID-19 patients compared to control group [12]. Other studies reported that 20% of COVID-19 patients might require rehospitalisation, especially in older individuals and those with comorbidities [18-20]. In contrast, two studies in Norway and Denmark that only included mild cases did not find a short-term increase in specialist care [13, 14, 21].

Although multiple studies have reported that the presence of sequelae after SARS-CoV-2 infection might explain the elevated healthcare use in the post-treatment period [12, 22], other factors might still exist. The example includes the exacerbation of prior comorbidities or newly diagnosed conditions caused by extensive examinations during patients' acute COVID-19 treatment episodes. For the latter, our findings of a short-term increase in utilisation indicated that, if this situation happened, the conditions were unlikely to be chronic.

Another important result from this study was the increased hospital utilisation before the treatment month. One possibility is that people with recent medical conditions were more likely to have more severe symptoms, hence increasing the risk of requiring hospital treatment [23]. The second one is the limited testing capacity and clear clinical guide in the early phase of the pandemic in Indonesia. This situation could lead individuals to visit healthcare facilities multiple times before being treated as confirmed COVID-19 cases [24-26].

This study also found a substantial variation with respect to the estimated changes in hospital costs across individuals treated in different calendar months. For example, during the month of treatment, patients treated in May 2020, on average, spent 169 million Rupiahs more than the control group. This figure decreased consistently with an estimated difference of about 138 million, 108 million, and 98 million Rupiahs among those treated in June, July, and August,

respectively. Broadly, COVID-related claims in Indonesia during this period were based on a fixed per day payment scheme with values depending on the type of services (i.e. requiring ICU with or without a ventilator, negative pressure isolation room, or regular isolation room) and patient's comorbidities. In addition, the government also reimbursed hospital expenditure on personal protective equipment (PPE) [27, 28]. Therefore, any gaps in costs between individuals treated in different calendar months might reflect changes in hospital resource usage. One plausible reason for this pattern is the rapid development of clinical guidelines for COVID-19 management [29].

Topics for future study

Given our results, a more detailed analysis of the type of diagnosis and procedure, including the use of primary care, is needed to fully understand the specific cause and type of increased healthcare utilisation in Indonesia. This type of question requires linked hospital and primary care utilisation, similar to what have been done in high-income countries [12, 13, 21, 22, 30]. The expanded database could also be utilised to examine the impact of less severe case of COVID-19 on healthcare utilisation.

In addition, since our study periods are mostly before the mass COVID-19 vaccination [31], a study using more recent data and a vaccination record is also needed. The observed patterns might change since the vaccination program is known to affect the course of COVID-19 disease. Lastly, due to the rapidly changing variants with different disease characteristics, data from different periods will be highly valuable in exploring the heterogeneity of its impact on healthcare utilisation.

Strengths and limitations of this study

To our knowledge, this study was among the first in low-and-middle-income countries (LMICs) that used a national database to examine the impact of COVID-19 infection (as reported in hospital claims data) on healthcare utilisations. Compared to self-reported measure as usually collected in surveys, the use of an administrative database would minimise recall bias.

However, we are also aware that our study has several limitations. First, the study population was limited to NHI members who had at least one regular hospital encounter before being treated with COVID-19. Second, our control group might be imperfect due to unmeasured factors that differentiate our COVID-19 group from the control group; hence residual

confoundings might still exist. We believe an improved dataset that covers all NHI members with more detailed individuals' characteristics might address some of these issues. Therefore, further cooperation with the Social Security Administrative Body of Health (BPJS-Kesehatan) and the Ministry of Health needs to be initiated. Third, our results around hospital costs are inherently sensitive to the change in clinical practice and the unit cost used for claim payments. Hence, our results should be carefully interpreted beyond the Indonesian context and the examined period.

Policy implication

This study provided evidence of a short-term elevation in utilisation of hospital services before and after COVID-19 hospitalisation in Indonesia that can be used for future health resource planning. This finding particularly important since the government of Indonesia is planning to shift the budget from the centrally allocated COVID-19 emergency fund to the regular NHI fund once the pandemic ends. As a social health insurance program that combines a subsidised scheme for the poor and a non-subsidised scheme based on a contributory mechanism for the non-poor (either through salary deduction or monthly premiums), it needs to anticipate the total cost of treating COVID-19 patients is required to keep its financial balance. For example, an additional government budget might be needed when a future outbreak arises and the estimated total costs of providing COVID-19 treatment exceed the available NHI fund.

Conclusion

Compared to the control group, individuals with COVID-19 that required hospital treatment appeared to have a higher utilisation of outpatient and inpatient services a few months before and after the month of treatment. Consequently, the costs of hospital services among these individuals were also significantly higher during these periods. These findings are essential for policymakers to understand the full impact of COVID-19 infection on healthcare resource utilisation, especially in more resource-constrained settings.

Acknowledgement

We would like to thank the Social Security Administrative Body of Health (BPJS-Kesehatan) team for providing the data used in this study.

Competing interest: None declared

Funding: This study is fully funded by Indonesia's Social Security Administrative Body of Health (BPJS-Kesehatan). Award/grant number 456/BA/0621.

Data sharing statement: the data is managed by the Social Security Administrative Body of Health (BPJS-Kesehatan) and only available on a reasonable request

Ethical clearance: This study obtained ethical clearance from the Ethical Committee of the Faculty of Medicine, Public Health, and Nursing, Universitas Gadjah Mada No KE/FK/0945/EC/2021.

References

1. Cascella M, Rajnik M, Aleem A, Dulebohn SC, Di Napoli R. Features, evaluation, and treatment of coronavirus (COVID-19). Statpearls [internet]. 2022.

2. Li J, Huang DQ, Zou B, Yang H, Hui WZ, Rui F, et al. Epidemiology of COVID-19: A systematic review and meta-analysis of clinical characteristics, risk factors, and outcomes. J Med Virol. 2021;93(3):1449-58. Epub 2020/08/14. doi: 10.1002/jmv.26424.

3. Stokes EK, Zambrano LD, Anderson KN, Marder EP, Raz KM, El Burai Felix S, et al. Coronavirus Disease 2019 Case Surveillance - United States, January 22-May 30, 2020. MMWR Morb Mortal Wkly Rep. 2020;69(24):759-65. Epub 2020/06/20. doi: 10.15585/mmwr.mm6924e2.

4. Hashim MJ, Alsuwaidi AR, Khan G. Population Risk Factors for COVID-19 Mortality in 93 Countries. J Epidemiol Glob Health. 2020;10(3):204-8. Epub 2020/09/22. doi: 10.2991/jegh.k.200721.001.

5. Taquet M, Dercon Q, Luciano S, Geddes JR, Husain M, Harrison PJ. Incidence, co-occurrence, and evolution of long-COVID features: A 6-month retrospective cohort study of 273,618 survivors of COVID-19. PLoS Med. 2021;18(9):e1003773. Epub 2021/09/29. doi: 10.1371/journal.pmed.1003773.

6. Chen C, Haupert SR, Zimmermann L, Shi X, Fritsche LG, Mukherjee B. Global Prevalence of Post COVID-19 Condition or Long COVID: A Meta-Analysis and Systematic Review. J Infect Dis. 2022. Epub 2022/04/17. doi: 10.1093/infdis/jiac136.

7. Ramzi ZS. Hospital readmissions and post-discharge all-cause mortality in COVID-19 recovered patients; A systematic review and meta-analysis. Am J Emerg Med. 2022;51:267-79. Epub 2021/11/16. doi: 10.1016/j.ajem.2021.10.059.

8. Humphreys H, Kilby L, Kudiersky N, Copeland R. Long COVID and the role of physical activity: a qualitative study. BMJ Open. 2021;11(3):e047632. Epub 2021/03/12. doi: 10.1136/bmjopen-2020-047632.

9. Nandasena H, Pathirathna ML, Atapattu A, Prasanga PTS. Quality of life of COVID 19 patients after discharge: Systematic review. PLoS One. 2022;17(2):e0263941. Epub 2022/02/17. doi: 10.1371/journal.pone.0263941.

10. Nopp S, Moik F, Klok FA, Gattinger D, Petrovic M, Vonbank K, et al. Outpatient Pulmonary Rehabilitation in Patients with Long COVID Improves Exercise Capacity, Functional Status, Dyspnea, Fatigue, and Quality of Life. Respiration. 2022;101(6):593-601. Epub 2022/02/25. doi: 10.1159/000522118.

11. Menges D, Ballouz T, Anagnostopoulos A, Aschmann HE, Domenghino A, Fehr JS, et al. Burden of post-COVID-19 syndrome and implications for healthcare service planning: A population-based cohort study. PLoS One. 2021;16(7):e0254523. Epub 2021/07/13. doi: 10.1371/journal.pone.0254523.

12. Ayoubkhani D, Khunti K, Nafilyan V, Maddox T, Humberstone B, Diamond I, et al. Post-covid syndrome in individuals admitted to hospital with covid-19: retrospective cohort study. BMJ. 2021;372:n693. Epub 2021/04/02. doi: 10.1136/bmj.n693.

13. Magnusson K, Skyrud KD, Suren P, Greve-Isdahl M, Stordal K, Kristoffersen DT, et al. Healthcare use in 700 000 children and adolescents for six months after covid-19: before and after register based cohort study. BMJ. 2022;376:e066809. Epub 2022/01/19. doi: 10.1136/bmj-2021-066809.

14. Skyrud KD, Hernaes KH, Telle KE, Magnusson K. Impacts of mild COVID-19 on elevated use of primary and specialist health care services: A nationwide register study from Norway. PLoS One. 2021;16(10):e0257926. Epub 2021/10/09. doi: 10.1371/journal.pone.0257926.

15. Agustina R, Dartanto T, Sitompul R, Susiloretni KA, Suparmi, Achadi EL, et al. Universal health coverage in Indonesia: concept, progress, and challenges. The Lancet. 2019;393(10166):75-102. doi: 10.1016/s0140-6736(18)31647-7.

16. Caniglia EC, Murray EJ. Difference-in-Difference in the Time of Cholera: a Gentle Introduction for Epidemiologists. Curr Epidemiol Rep. 2020;7(4):203-11. Epub 2021/04/02. doi: 10.1007/s40471-020-00245-2.

17. Wing C, Simon K, Bello-Gomez RA. Designing Difference in Difference Studies: Best Practices for Public Health Policy Research. Annu Rev Public Health. 2018;39:453-69. Epub 2018/01/13. doi: 10.1146/annurev-publhealth-040617-013507.

18. Lavery AM, Preston LE, Ko JY, Chevinsky JR, DeSisto CL, Pennington AF, et al. Characteristics of hospitalized COVID-19 patients discharged and experiencing same-hospital readmission—United States, March–August 2020. Morbidity and Mortality Weekly Report. 2020;69(45):1695.

19. Donnelly JP, Wang XQ, Iwashyna TJ, Prescott HC. Readmission and Death After Initial Hospital Discharge Among Patients With COVID-19 in a Large Multihospital System. JAMA. 2021;325(3):304-6. Epub 2020/12/15. doi: 10.1001/jama.2020.21465.

20. Iannaccone S, Alemanno F, Houdayer E, Brugliera L, Castellazzi P, Cianflone D, et al. COVID-19 rehabilitation units are twice as expensive as regular rehabilitation units. J Rehabil Med. 2020;52(6):jrm00073. Epub 2020/06/10. doi: 10.2340/16501977-2704.

21. Lund LC, Hallas J, Nielsen H, Koch A, Mogensen SH, Brun NC, et al. Post-acute effects of SARS-CoV-2 infection in individuals not requiring hospital admission: a Danish population-based cohort study. The Lancet Infectious Diseases. 2021;21(10):1373-82. doi: 10.1016/s1473-3099(21)00211-5.
22. Cohen K, Ren S, Heath K, Dasmarinas MC, Jubilo KG, Guo Y, et al. Risk of persistent and new clinical sequelae among adults aged 65 years and older during the post-acute phase of SARS-CoV-2 infection: retrospective cohort study. BMJ. 2022;376:e068414. Epub 2022/02/11. doi: 10.1136/bmj-2021-068414.
23. Gao YD, Ding M, Dong X, Zhang JJ, Kursat Azkur A, Azkur D, et al. Risk factors for severe and critically ill COVID-19 patients: A review. Allergy. 2021;76(2):428-55. Epub 2020/11/14. doi: 10.1111/all.14657.
24. van Empel G, Mulyanto J, Wiratama BS. Undertesting of COVID-19 in Indonesia: what has gone wrong? J Glob Health. 2020;10(2):020306. Epub 2020/10/29. doi: 10.7189/jogh.10.020306.
25. Ariawan I, Jusril H. COVID-19 in Indonesia: where are we? Acta Medica Indonesiana. 2020;52(3):193.
26. Djalante R, Lassa J, Setiamarga D, Sudjatma A, Indrawan M, Haryanto B, et al. Review and analysis of current responses to COVID-19 in Indonesia: Period of January to March 2020. Prog Disaster Sci. 2020;6:100091. Epub 2020/04/01. doi: 10.1016/j.pdisas.2020.100091.
27. Nugraha RR, Pratiwi MA, Al-Faizin RE, Permana AB, Setiawan E, Farianty Y, et al. Predicting the cost of COVID-19 treatment and its drivers in Indonesia: analysis of claims data of COVID-19 in 2020-2021. Health Econ Rev. 2022;12(1):45. Epub 2022/09/01. doi: 10.1186/s13561-022-00392-w.
28. Ministry of Health. PETUNJUK TEKNIS KLAIM PENGGANTIAN BIAYA PERAWATAN PASIEN PENYAKIT INFEKSI EMERGING TERTENTU BAGI RUMAH SAKIT YANG MENYELENGGARAKAN PELAYANAN CORONAVIRUS DISEASE 2019. Jakarta2020.
29. Dagens A, Sigfrid L, Cai E, Lipworth S, Cheng V, Harris E, et al. Scope, quality, and inclusivity of clinical guidelines produced early in the covid-19 pandemic: rapid review. BMJ. 2020;369:m1936. Epub 2020/05/28. doi: 10.1136/bmj.m1936.
30. Whittaker HR, Gulea C, Koteci A, Kallis C, Morgan AD, Iwundu C, et al. GP consultation rates for sequelae after acute covid-19 in patients managed in the community or hospital in the UK: population based study. BMJ. 2021;375:e065834. Epub 2021/12/31. doi: 10.1136/bmj-2021-065834.

31. Arifin B, Anas T. Lessons learned from COVID-19 vaccination in Indonesia: experiences, challenges, and opportunities. Hum Vaccin Immunother. 2021;17(11):3898-906. Epub 2021/10/07. doi: 10.1080/21645515.2021.1975450.

Fig 1 Click here to access/download;Figure;Fig 1.tif

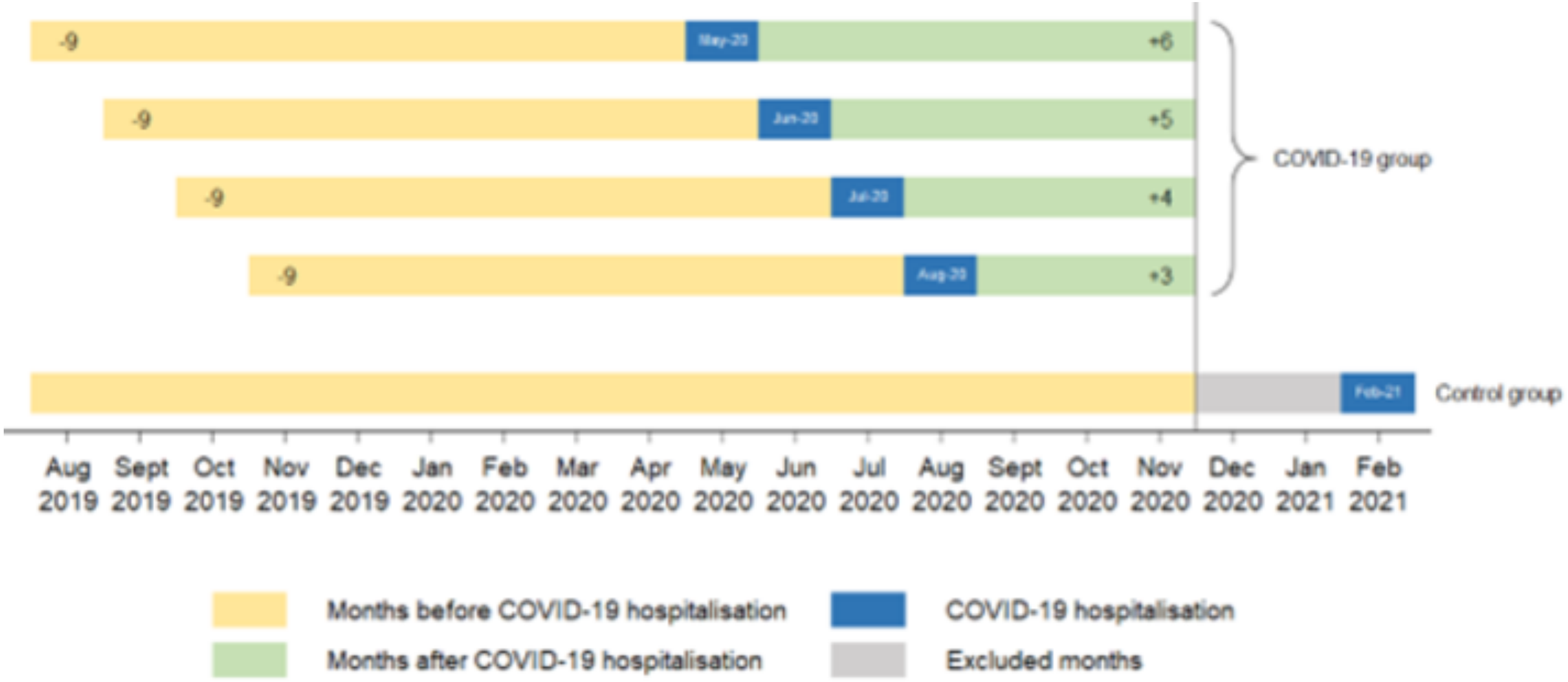

Fig 2 Click here to access/download;Figure;Fig 2.tif

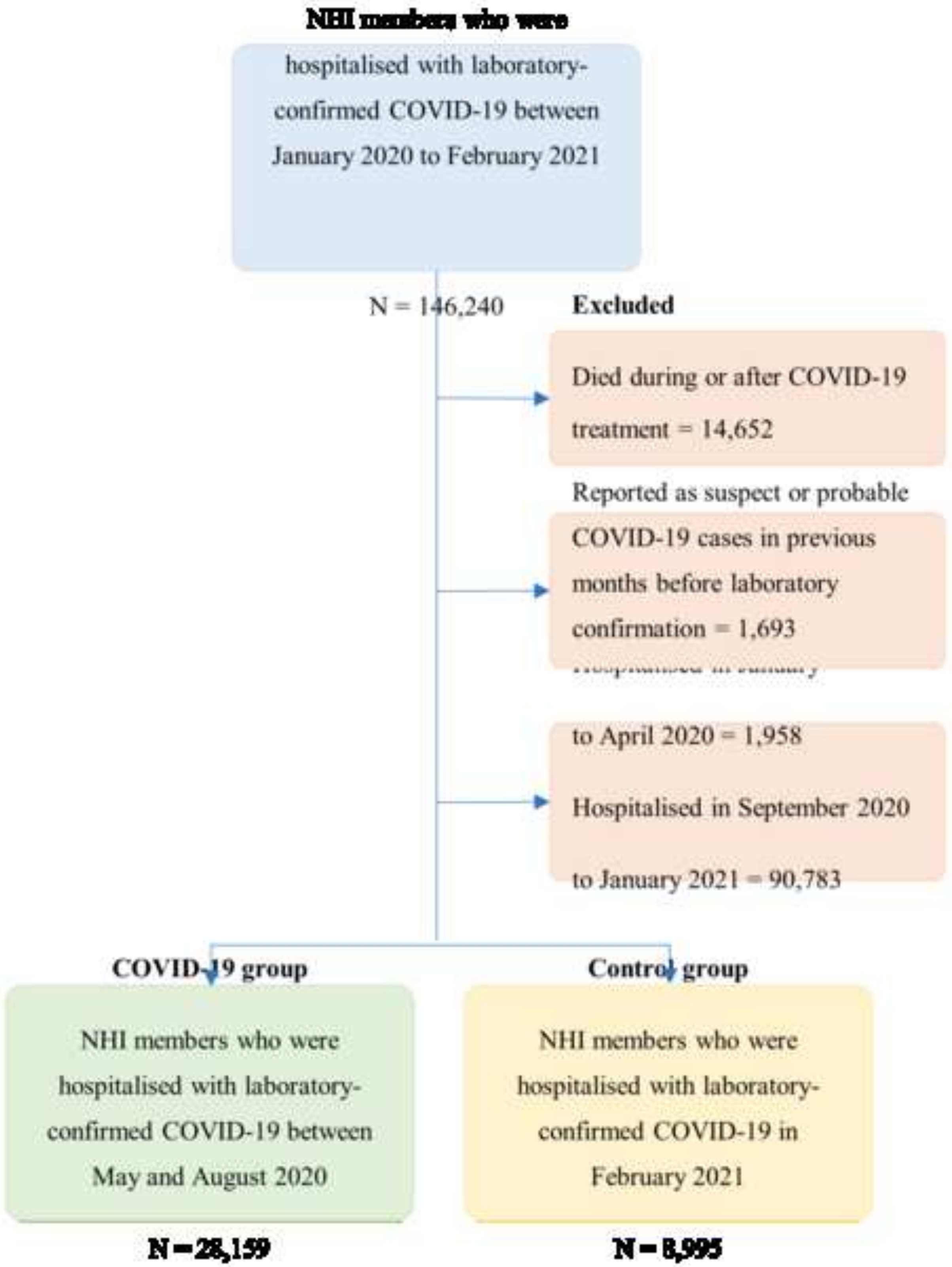

Click here to access/download;Figure;Fig 3.tif

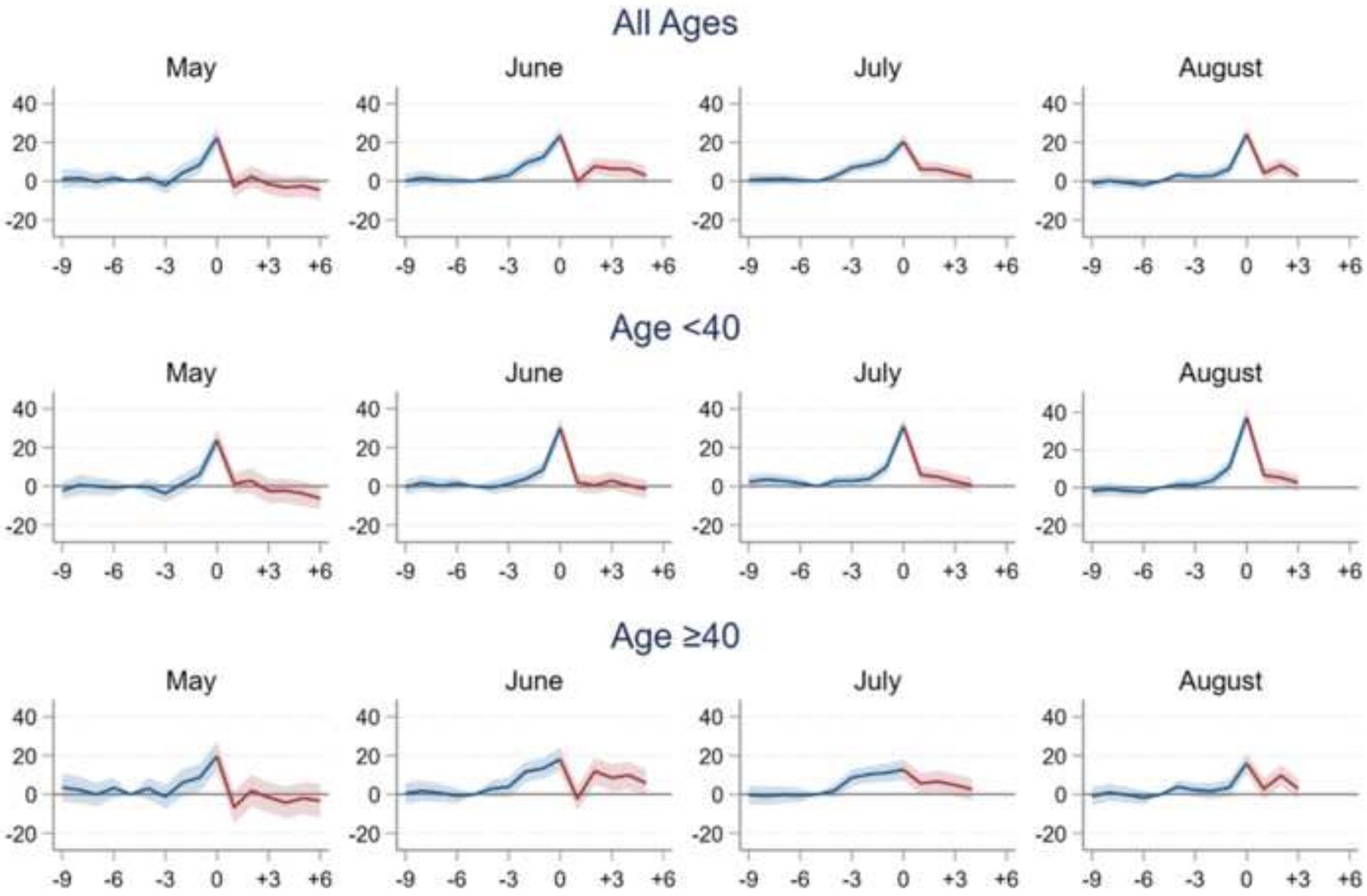

Fig 4

Click here to access/download;Figure;Fig 4.tif

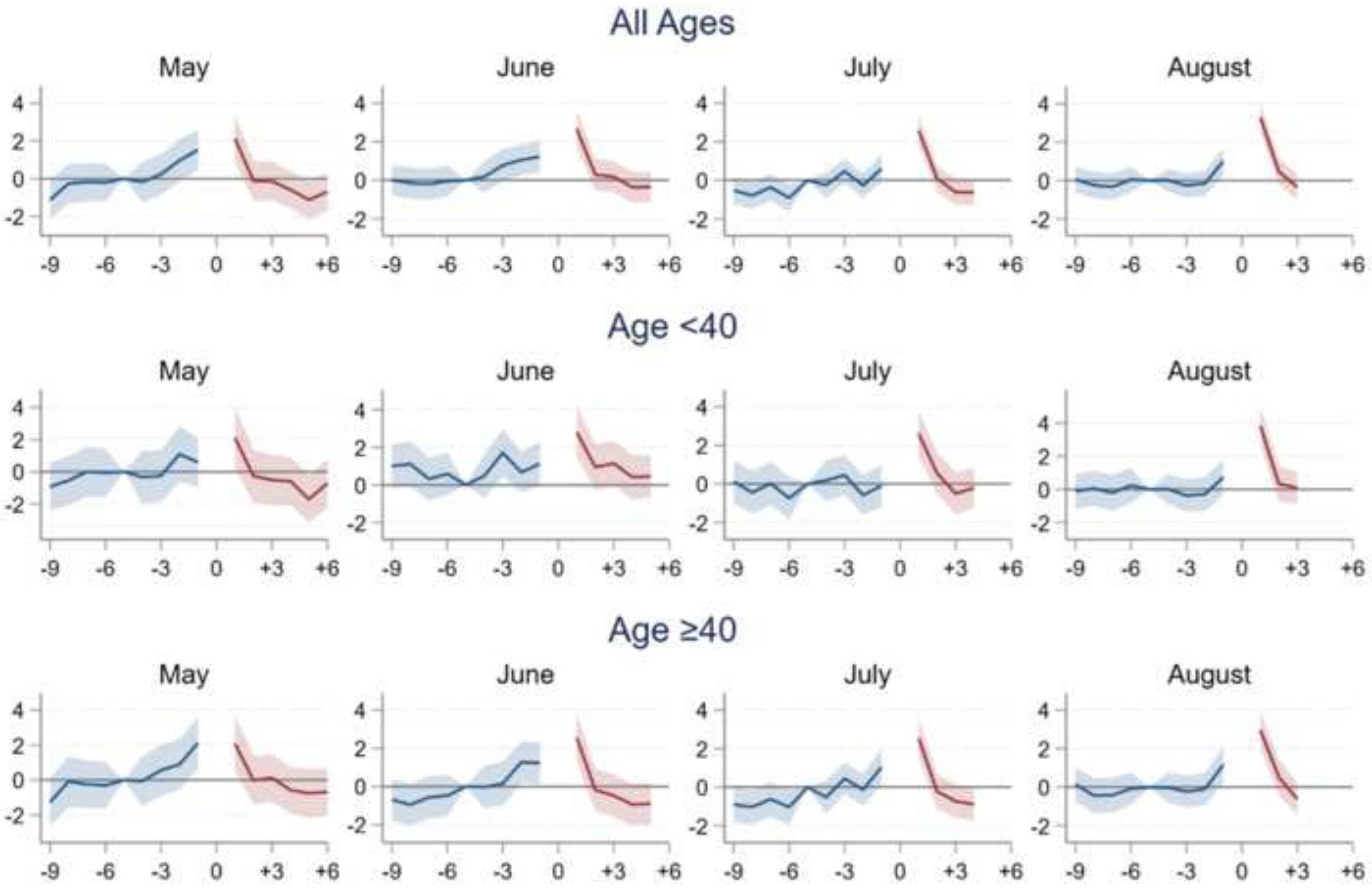

Supplement: Attachment — Submitted filename: PONE-D-22-31470_reviewer.pdf [file pone.0305835.s003.pdf]
