# Supplementary material for: Hospital services utilisation and cost before and after COVID-19 hospital treatment: Evidence from Indonesia
Source: PLoS One. 2024 Jul 5;19(7):e0305835. doi: 10.1371/journal.pone.0305835 (PMC11226039; doi:10.1371/journal.pone.0305835)
Supplement: Attachment — Submitted filename: Responses to Reviewers.docx [file pone.0305835.s004.docx]

Dear Editor,

Thank you for considering our article for publication at PLOS One. We really appreciate the comments and suggestions from you and the reviewers.

**Editor**
Journal Requirements:

When submitting your revision, we need you to address these additional requirements.

1. Please ensure that your manuscript meets PLOS ONE's style requirements, including those for file naming. The PLOS ONE style templates can be found at

<https://journals.plos.org/plosone/s/file?id=wjVg/PLOSOne_formatting_sample_main_body.pdf>

and

<https://journals.plos.org/plosone/s/file?id=ba62/PLOSOne_formatting_sample_title_authors_affiliations.pdf>

Thank you for the reminder. We have read the requirements and edited our files accordingly.

2. Please provide additional details regarding participant consent. In the ethics statement in the Methods and online submission information, please ensure that you have specified (1) whether consent was informed and (2) what type you obtained (for instance, written or verbal, and if verbal, how it was documented and witnessed). If your study included minors, state whether you obtained consent from parents or guardians. If the need for consent was waived by the ethics committee, please include this information.

If you are reporting a retrospective study of medical records or archived samples, please ensure that you have discussed whether all data were fully anonymized before you accessed them and/or whether the IRB or ethics committee waived the requirement for informed consent. If patients provided informed written consent to have data from their medical records used in research, please include this information.

We have added in Methods section Ethical Clearance with the sentence as follows,

“All data were fully anonymized before being accessed.”

3. Thank you for stating the following financial disclosure:

"This study is fully funded by Indonesia’s Social Security Administrative Body of Health (BPJS-Kesehatan). Award/grant number 456/BA/0621"

Please state what role the funders took in the study.  If the funders had no role, please state: "The funders had no role in study design, data collection and analysis, decision to publish, or preparation of the manuscript."

If this statement is not correct you must amend it as needed.

Please include this amended Role of Funder statement in your cover letter; we will change the online submission form on your behalf.

We have put the additional information in the funding section as mentioned in the cover letter

"The funders had no role in study design, data collection and analysis, decision to publish, or preparation of the manuscript.”

4. Your ethics statement should only appear in the Methods section of your manuscript. If your ethics statement is written in any section besides the Methods, please move it to the Methods section and delete it from any other section. Please ensure that your ethics statement is included in your manuscript, as the ethics statement entered into the online submission form will not be published alongside your manuscript.

We have moved the ethics statement to the Methods section.

5. In your Data Availability statement, you have not specified where the minimal data set underlying the results described in your manuscript can be found. PLOS defines a study's minimal data set as the underlying data used to reach the conclusions drawn in the manuscript and any additional data required to replicate the reported study findings in their entirety. All PLOS journals require that the minimal data set be made fully available. For more information about our data policy, please see <http://journals.plos.org/plosone/s/data-availability>.

Upon re-submitting your revised manuscript, please upload your study’s minimal underlying data set as either Supporting Information files or to a stable, public repository and include the relevant URLs, DOIs, or accession numbers within your revised cover letter. For a list of acceptable repositories, please see <http://journals.plos.org/plosone/s/data-availability#loc-recommended-repositories>. Any potentially identifying patient information must be fully anonymized.

Important: If there are ethical or legal restrictions to sharing your data publicly, please explain these restrictions in detail. Please see our guidelines for more information on what we consider unacceptable restrictions to publicly sharing data: <http://journals.plos.org/plosone/s/data-availability#loc-unacceptable-data-access-restrictions>. Note that it is not acceptable for the authors to be the sole named individuals responsible for ensuring data access.

We have updated our data availability statement to “All relevant data are within the manuscript and its Supporting Information files.” The relevant data are now submitted as supporting file.

6. Please upload a new copy of Figure xxxx as the detail is not clear. Please follow the link for more information: <https://blogs.plos.org/plos/2019/06/looking-good-tips-for-creating-your-plos-figures-graphics/> " <https://blogs.plos.org/plos/2019/06/looking-good-tips-for-creating-your-plos-figures-graphics/>.

we have reuploaded our figures to follow the requirements with the maximum quality we could produce.

Please use the space provided to explain your answers to the questions above. You may also include additional comments for the author, including concerns about dual publication, research ethics, or publication ethics. (Please upload your review as an attachment if it exceeds 20,000 characters)

**Reviewer 1**

This paper presents important information on hospital services utilization and cost before and after COVID-19 hospital treatment. The article is well written but there is some minor editing required. Also, I have highlighted a few areas of concerns in the submitted manuscript (see uploaded attachment.

Thank you for the comments and suggestions. We have addressed all of them in the revised manuscript. Here the detail:

1. Introduction, Paragraph 1:

- We have replaced the sentence “*A study in the US also mentioned that COVID-19 hospitalisation among patients with preexisting comorbidities was six times higher compared to those without ones*” with a new sentence “*A study conducted in Unites Stated found that COVID-19 hospitalization was six times higher in patients with preexisting comorbidities than in those without*”
- We edited the last sentence and delete the sentence of “*The same pattern also emerged from* ***multiple*** *settings, including in low-and-middle-income countries (LMICs),”*

2. Introduction, paragraph 2:

- We have deleted the word “also” in the first sentence
- We edited the sentence “*Due to its multi-organ involvement, some COVID-19 symptoms can persist up to six months after infection remission or more, defined as long covid or post-covid syndrome”* with a new sentence, *“Due to its multi-organ involvement, some COVID-19 symptoms can persist up to six months after infection remission or more,* ***which*** ***is*** *defined as long covid or post-covid syndrome”*

3. Methods, Section Study Population

We have added the comma after the word care in the sentence as follows

“*Individuals who were diagnosed based on RT-PCR positive results and treated, either in outpatient care or admitted to inpatient* ***care,*** *from May to August 2020 were included in the COVID-19 group.”*

4. Figure 1’s Note

We have moved the note to the Study Period’s paragraph as follows,

*“We considered a period of August 2019 to November 2020 in our analysis. December 2020 and January 2021 were excluded due to the possibility of elevated healthcare utilisation two months before individuals in the control group were hospitalised with COVID-19 in February 2021. Hence, we had a maximum of six months (June 2020 to November 2020) and a minimum of three months (September to November 2020) post-COVID-19 follow-up. For pre-COVID period, we included nine months before COVID-19 hospital treatment. The grouping strategy and the period of observation used in our analyses is shown in Fig 1”*

5. Figure 2’s Note

We have moved the note to the Study Participants’ paragraph as follows,

*“Figure 2 depicts the flow of study participants and their classification into COVID-19 and control group. “*

6. Result, Section Changes in Utilisation of Hospital Services

We have deleted the sentence “*The fifth month leading to the COVID-19 event was used as the reference period. We assumed the difference in outcome between the case and control in other periods to be constant”* and put this explanation in the Methods section Study Population as follows:

*“Those who treated in hospitals with COVID-19 in February 2021 were classified as the control group, assuming they never contracted COVID-19 in 2020. Note that in the NHI database, only results from RT-PCR (Reverse Transcriptase Polymerase Chain Reaction) done by Ministry of Health-accredited laboratories are used.”*

7. Results, Section Changes in Total Cost of Hospital Services

We have deleted the sentence, “In our regression, we used the period of 6 to 4 months before COVID-19 hospitalisation as the reference time where we assume no difference in admissions rate between the COVID-19 and the control group. We also lumped together 9 to 7 months before COVID19 to see whether there was any gap in outcomes between the two groups. “

The explanation related to the sentence has been included in the Methods, Section Study Period:

*“We considered a period of August 2019 to November 2020 in our analysis. December 2020 and January 2021 were excluded due to the possibility of elevated healthcare utilisation two months before individuals in the control group were hospitalised with COVID-19 in February 2021. Hence, we had a maximum of six months (June 2020 to November 2020) and a minimum of three months (September to November 2020) post-COVID-19 follow-up. For pre-COVID period, we included nine months before COVID-19 hospital treatment. The grouping strategy and the period of observation used in our analyses is shown in Fig 1”*

8. Discussion, Section Main Findings

- We have deleted this sentence, “*Among 28,155 Indonesian National Health Insurance (NHI) enrollees who were in hospitals with laboratory-confirmed COVID-19 between May and August 2020”*
- We revised the sentence *“Specifically, we found an increase outpatient utilisation that happened three months before and up to three months after the treatment and an uptick in admissions rate one month before and after the treatment”* with the additional word suggested by the reviewer as follows,

*“Specifically, we found an increase* ***in*** *outpatient utilisation that happened three months before and up to three months after the treatment and an uptick in admissions rate one month before and after the treatment.”*

- For the sentences related to the literature review, we have added some explanatory sentences to contrast their findings with ours, as follows

*“Other studies reported that 20% of COVID-19 patients might require rehospitalisation, especially in older individuals and those with comorbidities [21-23]. This finding was consistent with our results where higher post-COVID utilisation was observed mainly in the older patients. In contrast, two studies in Norway and Denmark that only included mild cases did not find a short-term increase in specialist care [14, 15, 24].*

9. Discussion, Section Policy Implication

We have replaced the word “elevation” with “to increase”

10. Conclusion

We have revised our conclusion not to recite our results. Therefore, our conclusion has been written as follows,

# *“Compared to the control group, individuals with COVID-19 that required hospital treatment appeared to have a higher utilisation of outpatient and inpatient services a few months before and after the month of treatment. This finding indicated that people who recently used more healthcare services were in a higher likelihood of contracting with COVID, either due to the worse health condition to begin with or because of the increasing contact with infected patients in the healthcare settings. More research is needed to fully understand the possibility of predicting COVID-19 infection based on prior healthcare utilisation. Lastly, the post-COVID pattern also need to be considered by policymakers when estimating the full impact of COVID-19 infection on healthcare resource utilisation, especially in more resource-constrained settings.”*

**Reviewer 2**

This is an informative and well written article. There are a number of suggestions for strengthening the content and findings.

Thank you for your comments and suggestions. We have addressed all of them and provide details of our responses below.

1. In the introduction and discussion, I would like to see more information on the population based epidemiology of covid-19 for Indonesia. This will help to better understand the landscape at this time. This study occurred during the first surges globally and the incidence and prevalence of the condition and other demographic characteristics of those effected and unaffected would be useful to see.

In the introduction, we have added the following sentence to briefly lay out the epidemiology of COVID-19 in Indonesia, particularly in the earlier period.

“In Indonesia, about 12% of the hospitalised patients died in the first three months of the pandemic, with higher rate among older people, male, and those with multiple comorbidities [4]. A more recent study indicated that districts with higher proportion of elderly and lower healthcare capacity had higher rate of COVID-19 mortality [5].”

2. The data is based upon a large administrative data base from Indonesia subjects included "with 28,159 Indonesian NHI enrollees treated with laboratory-confirmed COVID-19, compared with 8,995 individuals never diagnosed with COVID-19 in 2020." The methods used for laboratory confirmed Covid-19 dx is not described in detail. Was this according to the WHO criteria for diagnosing Covid-19. In addition, as this was based upon national data, were the laboratory methods across the country uniform and standardized?

We have clarified this issue in our revised manuscript by adding the following sentence in our method section.

“Note that in the NHI database, only results from RT-PCR (Reverse Transcriptase Polymerase Chain Reaction) done by Ministry of Health-accredited laboratories are used.”

3. More detail is needed for understanding the Covid-19 positive and "never diagnosed" groups. What is the level of comparability for the two groups (covid positive and "never diagnosed" being compared). Perhaps a propensity analysis would be useful to better understand the distance for all of the sociodemographic variables included to best understand the differences between the two groups compared. When matching I would base this on the Mahalanobis distance (MD), with the selection of the caliper. The study is only as good as an indepth understanding of the control group.

We agree that the comparability in characteristics between the Covid-19 group and the “never diagnosed” or control group is important. We have shown in Table 1 (and explained in the Result section) that there are several characteristics differences. However, we believe that we have addressed these observed variations by including them as control variables in our regressions. We further highlight our approach by revising our statistical analysis subsection.

In our results, we have also shown that parallel pre-trends – which is one way to indicate that our DiD was valid – were mostly satisfied. Therefore, we decide not to proceed with matching on characteristics before employing the DiD estimation. For example, we have now mentioned

“Our results indicated that up to the reference month, the differences in outcome between the COVID-19 and control group were close to zero and all statistically insignificant. This finding supported our assumption that the trend in outcome between the two groups were similar in the absence of COVID-19.”

4. For the control group, what is meant by "never diagnosed." Does this mean that those in the control group received the Covid-19 test and were negative? Is it possible that there was a fraction of individuals in the control group that were positive for covid?

The control group was the people that were diagnosed in February 2021, they have never been classified as positive/suspect/probable COVID-19 prior to their diagnosis in February 2021. We agree that there is some possibility that those individuals might be infected with COVID-19 beforehand. Those cases most likely to be asymptomatic. Otherwise, they would appear in the NHI dataset.

5. An instrumental variable analysis might be useful to better understand unmeasured variables that go uncontrolled for in the analysis? What might such unmeasured variables be?

We thank the reviewer for highlighting this issue. However, the use of instrumental variable is beyond the scope of this paper. Instead, we focus on expanding our discussion around the potential unmeasured confounding in our analyses. We have added the following sentences in the Strength and Limitation subsection.

“Second, our control group might be imperfect due to unmeasured time-varying factors that differentiate our COVID-19 group from the control group; hence residual confounding might still exist. For example, change in income or risk attitude – which was unmeasured in our dataset – might affect healthcare utilisation as well as the timing individuals were infected with COVID-19.”

5. As this study was done during the earlier surges of the global pandemic it would be useful to understand the severity of the presenting covid-19 and if data is available have a breakdown of costs and hospitalizations for those severely impacted.

The NHI data did not specifically classify the clinical severity of the presenting COVID-19 cases in Indonesia. The recorded “severity” level (1 to 3) is mainly used to reflect the intensity of hospital resource utilization during an inpatient stay used for reimbursement purpose.

We have added to Table 1 the distribution of those “severity” levels. We added the following sentence to reflect this addition.

“Based on the distribution of severity level, which reflects the intensity of hospital resource utilization, most of the patients were hospitalized when they were confirmed with COVID-19 infection, although with decreasing trend (96% in May 2020 and 89% in the control group)”

6. The focus for stratifications for hospital costs of services is by age (< 40 and >= 40). How was 40 years of age selected as the cutoff? Could more stratifications be included for age by a younger age group and the elderly? Results may be interesting. Can additional stratifications for other variables be included such as gender and educational level (a proxy for income).

We have added the reference to our choice of age cut-off in the Statistical Analysis section.

“Heterogeneity analysis was also conducted by splitting the sample into younger (<40 years old) and older (≥40) population, as well as by gender. The cut-off was considered given the risk of major comorbidities such as cardiovascular diseases and cancer known to increase exponentially after the age of 40.”

Unfortunately, we do not have information on income or educational level. We discussed the analyses by gender as the following.

“There were no substantial differences between males and female prior to month of treatment. However, the post-treatment trend indicated that males used more outpatient services after treated with COVID-19”

“There is no apparent gap between younger and older individuals or males and females with regards to inpatient utilisation as shown by the overlapping confidence interval in Fig 4.”

7. I would like to see the Indonesian currency "Rupiahs" converted to US dollars. Or at least a footnote indicating the conversion rate.

We have put the conversion in “Methods” section “Changes in Total Costs of Hospital Services” with the sentence as follows, “*Our estimates were presented in in thousand Indonesian Rupiah (US$1 equals to Rp14,500)”.*
