# Supplementary material for: Hospital services utilisation and cost before and after COVID-19 hospital treatment: Evidence from Indonesia
Source: PLoS One. 2024 Jul 5;19(7):e0305835. doi: 10.1371/journal.pone.0305835 (PMC11226039; doi:10.1371/journal.pone.0305835)
Supplement: Attachment — Submitted filename: Response to Reviewer Sept.docx [file pone.0305835.s005.docx]

Dear Asst. Prof. Dr. Nemer Badwan,

I am writing to express my gratitude for the second valuable feedback provided by the reviewers and yourself regarding our submitted manuscript titled "Hospital services utilisation and cost before and after COVID-19 hospital treatment: evidence from Indonesia." Here below our details comments and response:

**Journal Requirements**

Please review your reference list to ensure that it is complete and correct. If you have cited papers that have been retracted, please include the rationale for doing so in the manuscript text, or remove these references and replace them with relevant current references. Any changes to the reference list should be mentioned in the rebuttal letter that accompanies your revised manuscript. If you need to cite a retracted article, indicate the article’s retracted status in the References list and also include a citation and full reference for the retraction notice.

We have checked all references, and found no retracted references.

**Reviewers' comments**

Reviewer's Responses to Questions

Comments to the Author

1. If the authors have adequately addressed your comments raised in a previous round of review and you feel that this manuscript is now acceptable for publication, you may indicate that here to bypass the “Comments to the Author” section, enter your conflict of interest statement in the “Confidential to Editor” section, and submit your "Accept" recommendation.

Reviewer #1: All comments have been addressed.

Reviewer #3: (No Response)

2. Is the manuscript technically sound, and do the data support the conclusions?

The manuscript must describe a technically sound piece of scientific research with data that supports the conclusions. Experiments must have been conducted rigorously, with appropriate controls, replication, and sample sizes. The conclusions must be drawn appropriately based on the data presented.

Reviewer #1: Yes

Reviewer #3: Yes

3. Has the statistical analysis been performed appropriately and rigorously?

Reviewer #1: Yes

Reviewer #3: Yes

4. Have the authors made all data underlying the findings in their manuscript fully available?

The PLOS Data policy requires authors to make all data underlying the findings described in their manuscript fully available without restriction, with rare exception (please refer to the Data Availability Statement in the manuscript PDF file). The data should be provided as part of the manuscript or its supporting information, or deposited to a public repository. For example, in addition to summary statistics, the data points behind means, medians and variance measures should be available. If there are restrictions on publicly sharing data—e.g. participant privacy or use of data from a third party—those must be specified.

Reviewer #1: Yes

Reviewer #3: No

We have revised our data sharing statement to the following.

All relevant data used to produce the graphs in the paper are provided in S1 Table. The raw data that support the findings of this study are available from by Indonesia’s Social Security Administrative Body of Health (BPJS-Kesehatan), which were used under license for the current study, and so are not publicly available. Data is available from: <https://data.bpjs-kesehatan.go.id/> by applying via the website. Other researchers will be able to access the data set in the same way as the authors.

5. Is the manuscript presented in an intelligible fashion and written in standard English?

PLOS ONE does not copyedit accepted manuscripts, so the language in submitted articles must be clear, correct, and unambiguous. Any typographical or grammatical errors should be corrected at revision, so please note any specific errors here.

Reviewer #1: Yes

Reviewer #3: Yes

6. Review Comments to the Author

Reviewer #1: (No Response)

Reviewer #3:

**Reviewer 3**

The authors conducted a retrospective study on parameters related to COVID-19 healthcare in Indonesia. The idea to focus on periods before or after treatment of COVID-19 patients is highly interesting. Scope and idea of the study are clearly described. The study is relevant for understanding the pandemic’s impact of the pandemic on the healthcare system.

We thank the reviewer for the valuable comments. We have addressed each of your concerns below.

1. I suppose “COVID-19” here is defined as a lab-confirmed SARS-CoV-2 infection. Are information available that allow for identifying patients who actually developed corresponding respiratory systems? What might be the influence of patients without SARI?

Thank you for raising the issue that there might be some laboratory-confirmed SARS-CoV-2 infections that did not develop into Severe Acute Respiratory Infection (SARI). Unfortunately, we do not have information on symptoms or detailed treatment received by the patients to identify SARI. Therefore, classifying COVID-19 cases with and without SARI is not possible. We have added this limitation to our revised manuscript.

*“Lastly, due to the lack of information on symptoms and detailed resource utilisation, we could not identify whether patients developed Severe Acute Respiratory Infection (SARI) when treated with laboratory-confirmed COVID-19 infection.”*

2. If “COVID-19” were indeed defined as SARS-CoV-2 infection, I would recommend including SARI as a comorbidity too.

Because we cannot identify SARI, we cannot include it as comorbidity in our analysis. Similar to your first point, we have added this limitation to our revised manuscript.

3. How does hospital service utilization enter the statistical models? Is it a binary variable or a count per month? What kind of model was used, logistic or Poisson regression? Please add more details.

Thank you for pointing out this issue. We are aware that the hospital service utilisation is a count data, representing the number of visits per individual per month. We have added the following sentence in our method section to clarify this concern:

*“Although some of our outcomes were count variables, linear modelling can still be an appropriate choice, hence we estimate Equation 1 using Ordinary Least Square (OLS).”*

*Rothbard, S., Etheridge, J.C. & Murray, E.J.* *A Tutorial on Applying the Difference-in-Differences Method to Health Data. Curr Epidemiol Rep (2023). https://doi.org/10.1007/s40471-023-00327-x*

4. Is a linear model used for total claims (costs)? Did you check the distribution of costs? Was the variable transformed before entering the model? In my experience, this variable is highly skewed. Please add more details.

We agree that the skewness of claims (costs) data exist. However, since our aims is to estimate the quantity in absolute terms (Indonesian Rupiahs), we do not transform the costs variable first. To address this concern, we have added the following sentence to our method section.

*“In addition,* *for costs outcome, we conducted additional analysis where the variable is first transformed into a log form because of the skewness of the data.”*

We then described the findings in our result section.

*“These results were similar when we used log-transformed value of the costs variables.”*

5. The model formula (line 146) contains two coefficients, alpha and beta. I suppose the regression estimated coefficients for the other covariates too. If this is true, please extend the formula.

We have extended the regression equation to clarify that we also estimated the coefficients of other covariates. The revised equation is written below:

$$Y_{it}=\alpha_{i}+\Sigma_{t=Min}^{t=Max} {\beta_{1}}_{t} Month_{t}\times Covid_{i}+\beta_{2}Covid_{i}+\beta_{3}Month_{t}+{\boldsymbol{\beta}_{\boldsymbol{4}}\boldsymbol{X}}_{\boldsymbol{i}}Month_{t}+{\boldsymbol{\beta}_{\boldsymbol{5}}\boldsymbol{X}}_{\boldsymbol{i}}+\varepsilon_{it}$$

6. The model formula appears to contain interaction terms between the month and several covariates (age, sex, comorbidities). Did the model also contain terms for main effects of these covariates?

Yes, it did. We have now clarified the confusion by expanding our regression equation, as we have mentioned in the point number 5. In this new equation, the coefficient for the main effects of the covariates were represented by $\boldsymbol{\beta}_{\boldsymbol{5}}$ vector. However, because these variables were used as control variables, we did not report and discuss the estimated coefficients further.

7. I would like to know whether the authors did conduct the analyses stratified for total, age groups, and sex.

Yes, we did. We have already mentioned in the section “Statistical Analysis” of our initial manuscript as the following:

*“Heterogeneity analysis was conducted* *by splitting the sample into younger (<40 years old) and older (≥40) population, as well as by gender.”*

We have also reported the results for total sample, splitting by age group as well as gender or sex.

8. The authors present the proportions of different comorbidities. Are these comorbidities derived from ICD codes?

ICD-10 codes of the primary and secondary diagnoses were used to define the comorbidities (see the list below)

| **Comorbidity** | **ICD-10 Codes** |
| --- | --- |
| Hypertension | I10 – I16 |
| Type 2 Diabetes Mellitus | E11 |
| Heart disease | I11, I125 |
| Tuberculosis | A15 to A19 |
| Asthma | J45 |
| COPD | J44 |
| Cancer | C0 – C9; D1 – D4 |
| Liver disease | K70 – K77 |
| Chronic Kidney Disease | I12, I13, and N18 |
