# Supplementary material for: Hospital services utilisation and cost before and after COVID-19 hospital treatment: Evidence from Indonesia
Source: PLoS One. 2024 Jul 5;19(7):e0305835. doi: 10.1371/journal.pone.0305835 (PMC11226039; doi:10.1371/journal.pone.0305835)
Supplement: Attachment — Submitted filename: Response to Reviewer Feb24.docx [file pone.0305835.s007.docx]

Dear Asst. Prof. Dr. Nemer Badwan,

I am writing to express my gratitude for the third valuable feedback provided by the reviewers and yourself regarding our submitted manuscript titled "Hospital services utilisation and cost before and after COVID-19 hospital treatment: evidence from Indonesia." Here below our details comments and response:

**Reviewers' comments**

**Reviewer #5**

Major comments:

- Choice of reference period? What is the rationale for using the fifth month for hospital utilization and the 4-6 months before for cost? If you had chosen one month prior to hospitalization, the results would have been different. Why is the period in February used as comparison group?

*This is an arbitrary decision. The point of reference period is to show that prior to the reference period, the differences in changes in outcomes over time is close to zero (the parallel pre-trend). This is a matter of presentation. Moreover, referencing Skyrud et al. (2021), no long-term elevation in healthcare use was observed in this period, supporting our timeframe choice for a balanced analysis.*

*February 2021 was selected as the control group to establish a COVID-19 baseline, excluding December 2020 and January 2021 due to potential elevated healthcare utilization before the comparison group were hospitalized.*

- Is the aim of the study to investigate the utilization and cost for the ‘patients with COVID’ or the ‘COVID-19 disease’ itself? See the following text from your paper: “ We explored the short- to medium-term change in the pre- and post-acute period of COVID-19 treatment to fully understand the extent to which COVID-19 might be associated with the use of hospital services, especially in a resource-constrained setting.”

*It is for patients with COVID especially those who are treated in hospital, either inpatient or outpatient, due to COVID-9 which was confirmed based on standardised laboratory test. To clarify the aim, we revised the sentence:*

*"Our study aims to examine the impact of COVID-19 by analysing both the direct effects on patients treated for the disease and the associated changes in hospital service utilization and costs. This dual perspective provides insights into the short- to medium-term adjustments within hospital services pre- and post-acute COVID-19 treatment periods, especially critical for understanding the pandemic's ramifications in resource-constrained settings."*

- I would like to include the crude rate of hospital utilization and cost, not only the estimated difference in the difference plot, to be sure of the assumption that the trend in outcome between the two groups was similar in the absence of COVID-19.

*Thank you for your suggestion. We have added Tables showing the crude rate of hospital utilisation and cost as supporting information in Table S2.*

- I also want you to include the figures on the difference in the estimate for cost analysis and not only for health care utilization to see if the pre-trend assumption also holds here.

*Thank you for the suggestion. However, we believe this is unnecessary as our attempt to show the parallel pre-trend has been shown in Tables 2 to 4. Specifically, we have compared the total cost of COVID-19 and the comparison group at the reference period of 4 to 6 months before hospital treatment with a period of 7 to 9 months before. Hence, the estimates for the pre-reference period (7 to 9 months before hospital treatment), shown in the first row of the table, have indicated parallel pre-trends.*

- In Figure 4 (in-patient, May period), I think the estimates of the difference are not zero in the months prior to the fifth month before COVID-19 treatment but rather increasing; this may be discussed in the Discussion section.

*As discussed previously, the choice of reference group is a matter of presentation. We believe this does not require further discussion.*

- Additionally, also you should also discuss what causes the increased use of health care and cost from 5 months before treatment; it is a long period, are these frail patients?

*The increased use of healthcare and costs from 5 months before treatment could suggest that patients with pre-existing conditions are more likely to experience severe COVID-19 outcomes, necessitating more extensive healthcare utilization. This period might capture healthcare interactions related to managing chronic conditions, which are exacerbated or become more complex in the lead-up to a COVID-19 diagnosis. The notion of frail patients being more susceptible to higher healthcare use and costs aligns with broader medical literature, indicating that individuals with compromised health status face greater risks and healthcare needs during infectious disease outbreaks. We added in the discussion part*

- And why are the patients older in the comparison group?

*We are not sure about this, has not found any evidence support. One possible explanation for the older age profile in the comparison group (those affected in 2021) could be related to the evolution of the pandemic and changes in virus transmission dynamics over time. Initially, efforts might have focused on protecting and isolating older individuals, potentially leading to lower infection rates among this group in 2020. As the pandemic progressed into 2021, with the emergence of new variants or changes in public health measures, older individuals may have become more susceptible or exposed, leading to a higher representation in the comparison group.*

- There are more people in the comparison group with the non-subsidized membership scheme; could this affect health care utilization?

*Indeed, the presence of more individuals under the non-subsidized membership scheme in the comparison group could impact healthcare utilization patterns. However, it's important to note that during the pandemic, the government covered all patients' treatment costs for COVID-19, regardless of whether they had JKN (Indonesia's National Health Insurance) or not. This policy likely influenced a surge in access to COVID-19 services, with many claims categorized under the non-subsidized scheme. We anticipate this unique situation may have mitigated potential disparities in healthcare access between different insurance membership categories during the pandemic*

Minor comments:

- Please use the comparison group instead of the control group since it is not an RTC.

*Thanks, we replaced all control group to comparison group*

- Fig 1, misspelled in legend yellow, Months before COVID-10 hospitalisation

*Thanks, we revised the misspelling.*

- Severity level: how are the different levels defined? Please include this information in the method section and in the table notation. Is this the same as the COVID-19 severity used as the control variable in the regression? And have all included patients with COVID-19 as their main cause of hospitalization, or could it also be that they were arbitrary was tested when admitted to the hospital?

*Severity levels in our study are defined based on the Indonesian Case-Based Groups (INA-CBGs) system, a case-mix payment system that utilizes a software grouper application. The severity level is influenced by complications and comorbidities, which are indicative of the resource intensity level required for treatment during the first treatment episode where patients were laboratory-confirmed. Yes, these severity levels are used as control variables in our regression analyses. All included patients had COVID-19 as their primary cause of hospitalization; the system ensures that the diagnosis is not arbitrary but based on rigorous clinical assessment upon admission.*

*We included the information in the method section and we had it in table notation*

- In figure 1, it is stated that "lines represented the estimated difference between the COVID-19 and 215 control groups, controlling for demographic characteristics," but the controlling variable is the same as stated in the tables: “Regressions control for individuals’ year of birth, gender, COVID-19 severity, NHI membership segment, comorbidities prior to 2020 interacted with the month indicator, as well as month and province fixed effects.” But in the data source section, the basic demographic characteristics included year of birth, sex, district and province, and type of NHI membership, but nothing about COVID-19 severity or comorbidity. Please indicate all controlling variables in the figure legends.

*We included in the data source section.*

- Please be consistent with the word, like for instance, province or district instead of region, and COVID-severity instead of only severity.

*Thanks, we revised the region into province*

- Use log-transformed values of the cost variables. What are the results from the additional analysis? I can’t see that Table 2 includes these results. “ These results were similar when we used the log-transformed value of the cost variables (see Table 2).”

*Apologies for any confusion caused regarding the log-transformation of cost variables. To clarify, the log-transformation of cost data was performed as part of our preliminary analysis to address data skewness, rather than as an additional, separate analysis. However, after tested it has similar results and easier to interpreted in non-log-transformed, ensuring a more accurate and meaningful statistical analysis. We revised the method section and deleted the results sentences.*

**Reviewer #6**

The study authors conducted an analysis comparing inpatient hospital visit rates, outpatient hospital visit rates, and hospital-associated costs associated with COVID-19-positive patients compared with controls who have not yet contracted COVID-19. I believe the study is methodologically sound, but this can be strengthened with more details regarding data sources and the statistical methods.

- Page 3 line 62: “with” instead of “which” or some rewording of this first sentence is needed

*Replaced. Thanks,*

- Page 6 line 128: “in in”

*Replaced. Thanks,*

- Page 9 line 184: range of age minimum is 44.2, not 44.6, if I’m reading this correctly

*Replaced. Thanks.*

- It took me a while, but after studying Figure 1 and looking at the results, I believe I understand the methods. Please confirm if I’m understanding this correctly. If I am, then I think the methods can be written differently to more simply explain what was done and why

o The control group (unexposed) contracted COVID-19 eventually. This fact is the reason why this group was chosen as a control (as a means to adjust for some confounders)

o The case group (exposed) contracted COVID-19 earlier (2020)

o The difference between exposed and unexposed was determined by time period (-9, -8, 0, 1, …, 6)

*Thanks for the suggestions. We revised in method section make it simpler way to explain.*

- We would expect, on average, that the cases and controls are similar with respect to demographic and clinical characteristics. However, this is generally not true as COVID-19 changed over time (e.g., virulence), immunity changed, and public health mandates changed as well. Standardized differences are not reported but are probably meaningfully different between cases and controls during the “pre” period. DiD methods often use propensity score methods to adjust for such factors. Why was this not considered? This is particularly of concern when, using age as an example

*We appreciate the reviewer's suggestion to employ propensity score methods (PSM) to adjust for differences between cases and controls. In anticipation of these concerns, we explored the application of PSM in our analysis. However, upon implementation, we encountered significant challenges: the confidence intervals of our estimates became notably wider, reducing the precision of our results. We think it is because the matching process necessitated a reduced sample size, potentially limiting the statistical power and generalizability of our findings. Moreover, we had already accounted for individual characteristics as control variables in our regression model, interacting these with the month variable to robustly address temporal variations in their effects on the outcomes.*

- It appears that there is a lack of parallel trends for the inpatient analysis (Figure 4).

*Yes correct, we explained in the results section.*

- It’s unclear how clustering was taken into account since the same patient can be found in the analysis multiple times. From the table captions, it seems like there was some adjustment, but I did not see this mentioned in the methods.

*Yes, it is true that the same patient may appear multiple times in our analysis due to the monthly clustering approach we adopted. As noted in our Tables, standard error were clustered at individual level.*

- An assumption was made on page 6 that “those who were treated in hospitals with COVID-19 in February 2021 were classified as the control group, assuming they never contracted COVID-19 in 2020," but it’s not clear why an assumption had to be made. Rather, couldn’t this have been verified from the data?

*The assumption that individuals treated for COVID-19 in hospitals in February 2021 had not contracted the virus in 2020 was made to ensure that this is the right comparison group. While theoretically possible to verify past infections, this information is not available in our datasets.*

- If coefficient 0.1t represents the month-specific DiD, is this what’s reported in Table 2? It isn’t clear from the table descriptor or the description of the results in the prose. For example, the paragraph beginning on page 17, line 270, does not mention interaction in the interpretation.

*Not sure about the coefficient 0.1t you mentioned, but it the coefficients represented the estimated differences in total costs of hospital services (in thousand Rupiahs). Thanks we revised the description including paragraph page 17, line 270.*

- It is not specified in the methods by which cases of COVID-19 were captured or the accuracy of this. Different countries may have had differential access to PCR testing over time, and inpatient testing may be more accurately documented than outpatient testing.

*In our study, COVID-19 cases were identified through positive RT-PCR test results, with tests conducted by Ministry of Health-accredited laboratories. We acknowledge the potential variability in access to PCR testing across different regions and times, as well as the possibility that inpatient testing may be more systematically documented than outpatient testing.*

- The descriptors of cost were not presented. What types of costs were captured?

*Our analysis encompassed direct healthcare-related costs in bundled payment, such as hospitalization, medication, and diagnostic tests, outpatient visits and follow-up care at health facilities based on the claim reimbursement. We amended the manuscript to include a clear type of cost were analysed.*

- I don’t think using prior medical history to predict COVID-19 infection should be mentioned in the conclusions.

*Thanks, deleted.*
