# Supplementary material for: Hospital services utilisation and cost before and after COVID-19 hospital treatment: Evidence from Indonesia
Source: PLoS One. 2024 Jul 5;19(7):e0305835. doi: 10.1371/journal.pone.0305835 (PMC11226039; doi:10.1371/journal.pone.0305835)
Supplement: Attachment — Submitted filename: Response to reviewer.docx [file pone.0305835.s008.docx]

**Reviewer 5**

Thanks for the answers to all my previous comments. I only have one additional comment.
Could you refer to all the S2 tables in the manuscript?

We have now added the following sentences to refer to the Supplement S2 Tables in the end of our Results Section.

“In addition to the event-study estimates presented above, for transparency, we also reported the raw mean of monthly outpatient utilisation, inpatient utilisation, and hospital costs among COVID-19 groups and the comparison group (for all individuals) in Supplement S2 Tables B1, B2, and B3, respectively.”

**Reviewer 7**

The construction of the treatment (covid-19) and comparison (not yet covid-19) is crucial for the interpretation of the results. The author states that “the comparison group comprised individuals treated for COVID-19 in hospitals in February 2021, under the presumption they had not contracted the virus in 2020” (line 115). This implies that what is estimated is not the impact (on subsequent utilization/costs) of having COVID-19 vs. not having COVID-19 in 2020, but the impact (on subsequent utilization/costs) of having COVID-19 in 2020 vs. having COVID-19 in 2021. This distinction has important implications for interpretation. For example, the authors’ DiD estimates how much higher the costs of treating a COVID-19 patient were in 2020 compared to 2021 (not the costs of treating a COVID-19 patient vs. not treating one). And, as another example, the authors do not shed any light on the presence of sequelae after SARS-CoV-2 infection, only on the question of whether possible sequelae are different in 2020 vs. 2021.

Clearly, these are two different questions (i. effects on utilization of COVID; ii. effects on utilization of COVID in 2020 vs. 2021), due, e.g., to the strain on health care services and other societal restrictions at the beginning of the pandemic. It seems obvious that the services’ ability to treat patients (both those with COVID-19 and other patients) was very different in 2020 than in 2021 (and definitely in 2024). It is crucial that the authors make this distinction between the two research questions clear to the readers (including in the abstract) and that they improve precision in how they describe their research question (and findings) throughout the paper (including considering making the title communicate this better).

In doing so, the authors may consider describing briefly other ways of constructing the comparison group and its implications for the research question, e.g., using contemporaneous patients with negative PCR tests (in 2020), like in reference (14), which would address the question of the impact of SARS-CoV-2 vs. no SARS-CoV-2 (instead of SARS-CoV-2 in 2020 vs. 2021). The authors should also be careful in pointing out the differences in research questions when comparing them with previous studies and when discussing policy implications (both in Discussion). This said, it appears to me that the question actually addressed by the authors' regressions (impact on utilization of COVID in 2020 vs. 2021) is of interest; it just needs to be stated and motivated clearly to the reader.

Thank you for your comment. In our Methods, we have made clear that the Comparison group, those who were treated with COVID-19 in February 2021 were assumed to not contracted COVID-19 in 2020. In the Study Population subsection, we mentioned “Those who treated in hospitals with COVID-19 in February 2021 were classified as the control group, assuming they never contracted COVID-19 in 2020.”

We have added the following sentence to elaborate our assumption.

"This assumption is plausible given reinfection rate within one year follow up was only about 5% [17].”

In Study Population subsection, we revised our explanation to address your concern.

“We considered a period of August 2019 to November 2020 in our analysis. During this period, the comparison group, those who were treated with COVID-19 in February 2021, was assumed to not yet contracted the disease."

We believe that this is now clear that in our post-COVID follow-up, our comparison group was realistically assumed to have not contracted COVID-19, hence appropriate as control group and our main research question and subsequent interpretation of the results remain.

It seems that those dying are excluded from the sample, instead of the more common approach of censuring them from the month of death: “Similarly, those who died during COVID-19 hospitalization or in subsequent months were also excluded from the analysis.” (line 114). If medical treatment for COVID-19 was more effective in 2021 than in 2020 (e.g., due to less strain on the services or some immunity due to low-dose exposure or vaccination (too early for that in Indonesia?)), this introduces a potentially serious bias to the analysis: The comparison group receives more utilization because they survive longer (which would lead to an underestimation of how much higher costs were in 2020 than in 2021). This problem is not easy to handle, but it absolutely deserves a serious and sincere discussion. It would also be important to inform the reader of the survival in the two groups (i.e., by providing death rates by 3-6 months or even the impact on death using the same regressions). Was survival measured for the same length of time in the comparison group?

Thank you for your comment. First, we would like to reiterate that the comparison group (who were treated with COVID-19 in February 2021) had not yet contracted COVID during our pre- and post-COVID periods among those treated with COVID-19 in May to August 2020 (the COVID-19 group). This means that during these follow-up periods, the comparison group was assumed to use healthcare services that were not associated with them having contracted COVID-19.

Second, our decision to focus on survivors was precisely to avoid the issue of reduced healthcare use of COVID-19 patients due to their higher risk of mortality following their hospital treatment. We added the following explanation to our Study Population subsection.

“This decision was made to focus on COVID-19 survivors, hence avoiding potential issue of reduced healthcare use by the COVID-19 group due to their higher post-hospital treatment mortality.”

Minor things.

I do not understand why “individuals who were under monitoring and recorded as suspect or probable cases of COVID-19 in the previous months before laboratory confirmation were excluded” (line 113), since it makes the relevance of the results restricted to those who are actually being tested (which I presume could depend on the severity of the infection or socioeconomic status, or, as noted by the authors, that the hospital/patient have access to PCRs done by a Ministry of Health-accredited laboratory). On the other hand, I do not see that this has important methodological problems (except that PCR testing capacity was presumably smaller relative to the number of infections in 2020 than in February 2021) as long as the reader understands that the population under study is narrow. However, in note to Table 1, it is stated that “individuals without any hospital encounters before January 2020 (..) were excluded.” How is this incorporated for the comparison group in a way that does not introduce differences/bias across the two groups?

We agree with your concern that this is part of the data limitations. We have mentioned that the narrowness of this study population in our study limitation subsection.

Regarding the exclusion of individuals without any hospital encounters before January 2020, we made a mistake in the note from previous iteration of the analysis. The current results did not make this exclusion. We have revised our note to Table 1 as the following.

“Individuals who died of any causes during or after COVID-19 treatment were excluded.”

It would be very informative if Table 1 also included the outcome variables (i.e., the raw out- and inpatient rates and costs by, e.g., 3 months) and a reference to Supplement Table 2 for details (but this supplement table is not informative about the comparison group since we are only informed about post-infection months for the treated (not the comparison)—please fix).

It would be difficult to provide the raw mean of our outcome variables in Table 1 for both COVID-19 and comparison group since the months that we used as pre- and post-COVID periods were different. In Figure 1, we have made it clear that, for example, in COVID-19 group who were hospitalised in May 2020, the post-COVID period that was analysed was June to November 2020.

The comparison of raw outcome means for COVID-19 groups and the comparison group were provided in S2 Tables. To make the Tables more informative, we have now revised the Table notes as the following.

“The shaded cells are the post-COVID period for each COVID-19 group. The underlined figures are the reference period for each COVID-19 group. A simple analysis is to compare the outcomes in the same months of the COVID-19 groups with the comparison group, adjusted with the difference in outcome between the two groups at the reference month (the underlined figure). For example, the post-COVID period for May 2020 COVID-19 group is June to November 2020 and the reference month is December 2019. Robust standard errors in parentheses.”

Eq. 1 includes individual fixed effect (alpha i), but also a dummy for being in the treatment group (Covid i). I presume there is a typo here, as the individual fixed effect would absorb all other time-invariant individual characteristics (and, obviously, being in the treatment group does not change over time).

Thank you for the correction. We have now deleted $\alpha_{i}$ from the equation since it is not estimated in our regression.

Given the vast population of Indonesia, some information on why there are so few people in the sample is warranted.

We have now revised the first limitation of the paper as the following.

“First, the study population was limited to NHI members who had at least one regular hospital encounter before being treated with laboratory-confirmed COVID-19. This made our sample smaller than the cumulative number of confirmed cases in Indonesia which were about 1.3 million cases as of February 2021.”

I don’t understand why the cost results are not also presented in a figure (like the nice figures for in- and outpatient results; please refer the reader to Supplement Table 1 in the note to these figures).

We did not present the cost results as Figures because the scale of the estimated effect of COVID-19 across period greatly differed. For example, in Table 2, the point estimate for May 2020 COVID-19 group at 1 month after COVID-19 hospitalisation was 2,453 (in thousand IDR) while the point estimate at the hospitalisation month was 168,649 (in thousand IDR). Because of this concern, presenting the results as Figures would not be as informative as detailed results provided in Tables 2-4.

I was not able to reach the data application site using the link in the data sharing statement.

We are aware about the difficulty of accessing BPJS Kesehatan website from overseas. This issue is, however, beyond our capacity as researchers.

“before” is lacking in line 31 of the abstract.

Thank you for pointing this out. We have now revised this.

Strictly speaking, the PCR test is for SARS-CoV-2 (i.e., the virus) and not for COVID-19 (the disease); the authors might consider their precision in the use of these two terms.

Thank you for the suggestion. We have clearly stated in our Study Population section that we define COVID-19 in our paper as PCR-confirmed COVID-19 cases. We believe that this narrower definition is appropriate to avoid too many clinical practice variations in COVID-19 diagnosis, especially in the early years of the pandemic.
